# Gene Transfection in High Serum Levels: Case Studies with New Cholesterol Based Cationic Gemini Lipids

**DOI:** 10.1371/journal.pone.0068305

**Published:** 2013-07-04

**Authors:** Santosh K. Misra, Joydeep Biswas, Paturu Kondaiah, Santanu Bhattacharya

**Affiliations:** 1 Department of Organic Chemistry, Indian Institute of Science, Bangalore, India; 2 Department of Molecular Reproduction, Development and Genetics, Indian Institute of Science, Bangalore, India; 3 Chemical Biology Unit of JNCASR, Bangalore, India; Albert-Ludwigs-University, Germany

## Abstract

**Background:**

Six new cationic gemini lipids based on cholesterol possessing different positional combinations of hydroxyethyl (-CH_2_CH_2_OH) and oligo-oxyethylene -(CH_2_CH_2_O)_n_- moieties were synthesized. For comparison the corresponding monomeric lipid was also prepared. Each new cationic lipid was found to form stable, clear suspensions in aqueous media.

**Methodology/Principal Findings:**

To understand the nature of the individual lipid aggregates, we have studied the aggregation properties using transmission electron microscopy (TEM), dynamic light scattering (DLS), zeta potential measurements and X-ray diffraction (XRD). We studied the lipid/DNA complex (lipoplex) formation and the release of the DNA from such lipoplexes using ethidium bromide. These gemini lipids in presence of a helper lipid, 1, 2-dioleoyl phophatidyl ethanol amine (DOPE) showed significant enhancements in the gene transfection compared to several commercially available transfection agents. Cholesterol based gemini having -CH_2_-CH_2_-OH groups at the head and one oxyethylene spacer was found to be the most effective lipid, which showed transfection activity even in presence of high serum levels (50%) greater than Effectene, one of the potent commercially available transfecting agents. Most of these geminis protected plasmid DNA remarkably against DNase I in serum, although the degree of stability was found to vary with their structural features.

**Conclusions/Significance:**

-OH groups present on the cationic headgroups in combination with oxyethylene linkers on cholesterol based geminis, gave an optimized combination of new genera of gemini lipids possessing high transfection efficiency even in presence of very high percentage of serum. This property makes them preferential transfection reagents for possible *in vivo* studies.

## Introduction

Gene therapy is a promising method in modern medicinal research, which employs “Gene as medicine” [1]. This line of treatment offers new hope for survival against many diseases which have genetic origins like cancer [2], diabetes [3], cystic fibrosis [4], AIDS [5] and cardiovascular diseases [6] etc. This strategy has broadened the scope of playing with the genetic material to avoid, remove or replace the fundamental cause of the diseases by delivering the desired genes or oligonucleotides or by blocking the ‘disease-causing’ sequence from transcription and translation. Towards this end, in the early phase of research, natural viruses were used as gene transporters [4]. Despite their high DNA delivery efficiency, viruses are sometimes inappropriate for the therapeutic applications. This is because viruses possess high risk of being infectious or adversely immunogenic [4], [7]–[9]. Accordingly a great deal of work has been carried out in the field of design and syntheses of non-viral gene transfection agents that do not elicit significant immunogenic reactions. Such non-viral gene transfer agents include pseudoglyceryl lipids [10]–[14], cholesterol derivatives [15]–[20], polymers [21], [22] and dendrimers [23], [24] etc. many of which have shown variable degree of cell viabilities as well as transfection efficiency. Of these the cationic lipid based DNA transfer agents turn out to be most attractive due to their amenability to structural modifications at the molecular level to improve the gene transfer efficiency. However, the presence of serum often severely reduces the transfection activity of the cationic lipid based reagents. Positive charge on the surfaces of cationic lipid/DNA complexes results in a non-specific adsorption of negatively charged plasma proteins in serum leading to the loss of transfection efficiency [25]. For this reason to develop a structure-activity relationship (SAR), most of the transfection experiments are performed in absence of serum in vitro. However, for effective transfection *in vivo*, one cannot escape high concentrations of blood serum in many cases. Clearly the surface charge of the lipoplexes plays a key role in determining their stability in serum.

Correlation of the physical chemical data with the *in vitro* transfection efficiency suggests that lipoplex instability [26]–[28], DNA release ability [29] and uptake efficiency [30] are “key factors” in transfection efficiency. However, while in low serum levels or in absence of serum, the aggregate instability imposed by helper lipid DOPE is advantageous, in contact with serum proteins, the dissociation of lipoplexes followed by aggregation often leads to precipitation and results in the loss of efficient transfection. The goal of the present work is to develop cationic lipids with variable spacer between two cationic headgroups, such that at optimal spacer and headgroup combination, good transfection activity of lipids is maintained in serum.

After the discovery of *3β-*[*N-(N’,N’*-dimethylaminoethane) carbamoyl] cholesterol (DC-Chol) [15], many cationic cholesterols were developed that showed efficient gene transfer activities [16]–[18]. Such molecules are made of a cholesteryl skeleton which is attached via a linker to the cationic headgroup. Both the linker and the nature of the cationic headgroup are important determinants of the gene transfer efficiency and cytotoxicity [16]. Thus the type of amine headgroups of such amphiphiles influenced the transfection efficiency [31]. Notably among various cationic headgroups, the ones possessing -CH_2_CH_2_OH manifested improved gene transfection ability than their counterparts that are devoid of -CH_2_CH_2_OH groups [32]–[34]. High levels of transfection were also reported from non-glycerol based cationic lipids with hydroxyethyl headgroups [35], [36].

**Figure 1:**
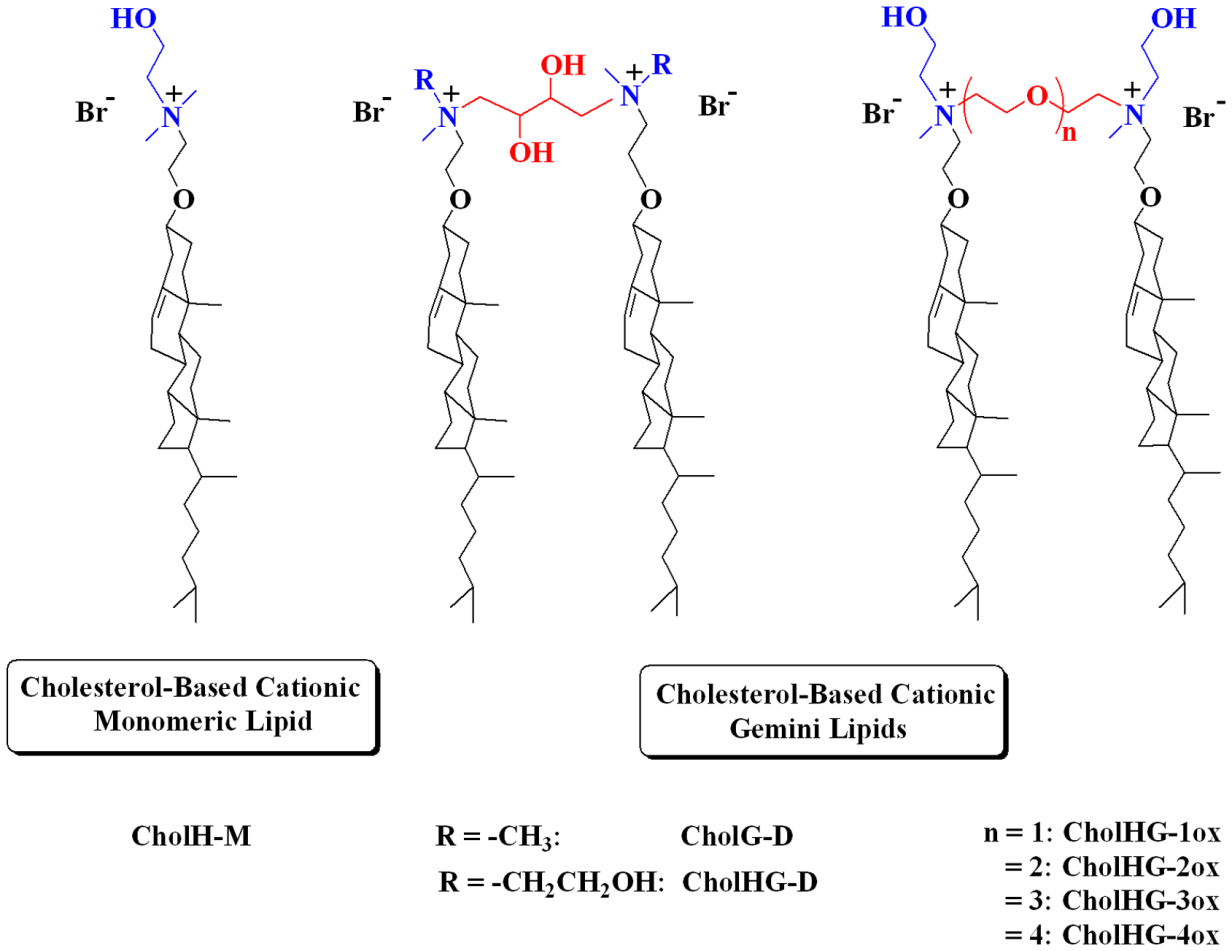
Molecules of interest. Molecular structures of the cholesterol-based cationic gemini lipids used in the present investigation.

Gemini lipid versions of monomeric cationic cholesterol compounds have recently shown significant improvements in the gene transfer properties than their monomeric counterparts [37]. However, the gene transfection properties of the corresponding dicationic gemini lipids possessing -OH group both at the headgroup and on the spacer segments are still not known. Towards this end, we present here a new set of synthetic geminis based on cholesterol bearing -OH groups both at the headgroup and at the spacer segment that connects the two cholesteryl units (Figure 1).

Each new cholesteryl lipid was dispersed in water and the corresponding aggregates were characterized using TEM to discern the morphologies formed from each of them in aqueous media, DLS to determine the hydrodynamic diameter and XRD study of the cast lipid films to determine the widths of the aggregates formed. These gemini lipids in presence of helper lipid, DOPE showed significant enhancements in the gene transfection activities as compared to their monomeric lipid counterparts. The corresponding gemini lipid/DOPE mixtures were also superior to many well known commercially available transfection agents. Moreover the gemini lipid based DOPE formulations did not show any significant level of toxicity at the concentrations at which transfections were performed. CholHG-1ox was found to be the most effective lipid even at very high serum concentration of 50% (vol/vol), and showed transfection activity greater than Effectene, one of the most effective commercial transfecting reagents. Hydroxyethyl groups present on the cationic cholesterol headgroups in combination with oxyethylene linker afforded an optimized combination of new genera of geminis which showed high transfection efficiency in presence of very high percentage of serum.

## Results and Discussion

### A. Synthesis

**Figure 2:**
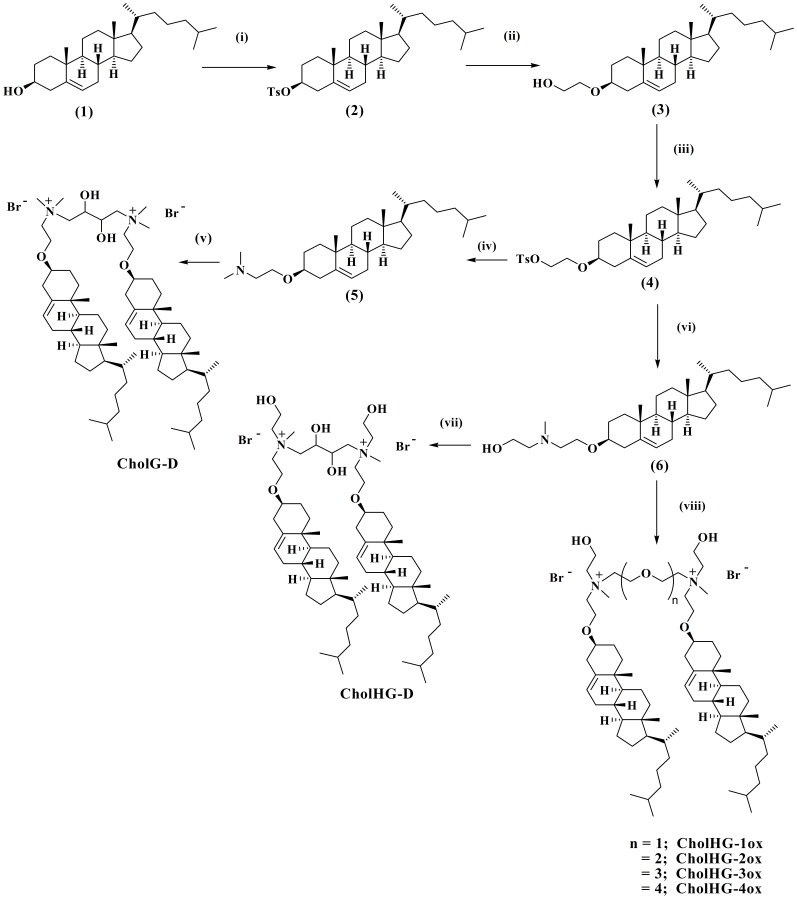
Reaction conditions and yields: (i) *p*-TsCl, Py, CHCl_3_, DMAP, 0°C, 6 h, 91.5%; (ii) Ethylene glycol, dry dioxane, 4 h, reflux, 80%; (iii) *p*-TsCl, Py, CHCl_3_, 0°C, 6 h, 88%; (iv) Dimethylamine, MeOH, screw-top pressure tube, 80°C, 24 h, 99%; (v) 1,4-Dibromobutane-2,3-diol, MeOH-EtOAc (4 mL, v/v: 1/1), screw-top pressure tube, 80°C, 48–72 h, 48%; (vi) *N*-Methylethanolamine, CH_3_CN, 24 h, reflux, 97%; (vii) 1,4-Dibromobutane-2,3-diol, MeOH-EtOAc (4 mL, v/v: 1/1), screw-top pressure tube, 80°C, 48–72 h, 41%; (viii) α, *ω*-dibromoalkoxyalkane, MeOH-EtOAc (4 mL, v/v: 1/1), screw-top pressure tube, 80°C, 48–72 h, 40–50%.

Six new cholesterol-based cationic gemini lipids differing in their headgroups and the spacers that connect the two headgroups were synthesized (Figure 2). Each new cholesterol-based cationic gemini was fully characterized by ^1^H-NMR, ^13^C-NMR, mass spectrometry, and CHN analysis, *cf.* experimental section.

### B. Physical Characterizations

#### Transmission Electron Microscopy (TEM)

**Figure 3:**
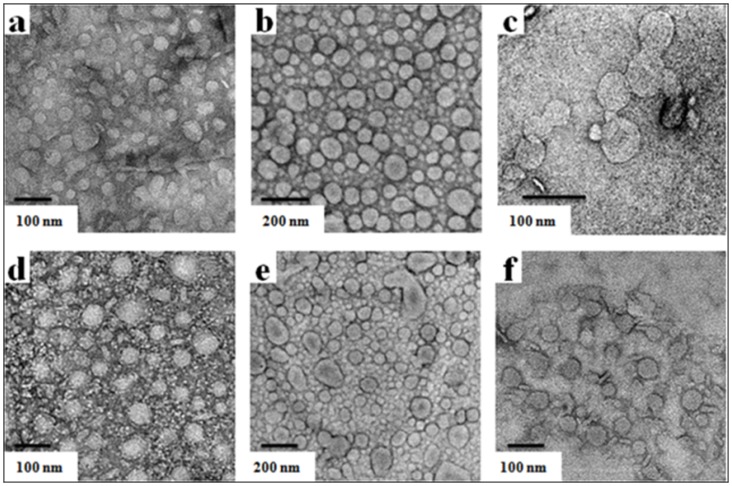
Transmission electron microscopy. Negative-stain transmission electron micrographs of aqueous suspensions of the cholesterol-based cationic geminis (a) CholG-D, (b) CholHG-D, (c) CholHG-1ox, (d) CholHG-2ox, (e) CholHG-3ox and (f) CholHG-4ox.

TEM of each lipid suspension upon negative-staining revealed that each of the cholesterol-based gemini formed closed vesicular aggregates in aqueous media as shown in the Figure 3. From TEM experiment, we observed that the diameters of these cholesterol-based cationic gemini aggregates ranged from 30 to 130 nm in diameter. TEM studies further indicate that CholHG-1ox gives liposomes of lowest in size whereas CholHG-3ox affords liposomes that are largest in size. In certain cases (CholHG-D and particularly CholHG-1ox), the aggregates in such micrographs appear to remain ‘connected’ to each other and this could be due to the hydrogen bonding interactions among the hydroxyl groups attached to the headgroup or in the spacer. Reduction in the sizes observed under TEM could be because of the shrinkage of the aggregates induced while drying before taking TEM images [38]. Representative negative-stain TEM images of aqueous suspensions of lipoplexes of CholHG-1ox, CholHG-3ox and CholHG-D at optimized lipid/DOPE and N/P ratio are shown in the Figure S1.

#### Dynamic Light Scattering (DLS)

DLS data (Table 1) show that the hydrodynamic diameters of these lipid aggregates in aqueous media ranged from ∼137–220 nm in diameter. From the DLS studies, we further observe that among the cholesterol-based geminis with oxyethylene spacer, CholHG-3ox aggregates are the largest (diameter ∼220 nm) whereas the CholHG-1ox aggregates are the smallest (diameter ∼137 nm). Among the geminis with OH group in the spacer, we observe that CholHG-D (having hydroxyl both at the headgroup and at the spacer) has the hydrodynamic diameter of ∼212 nm whereas CholG-D (having hydroxyl function only at the spacer) has smaller diameter of ∼147 nm. The hydrodynamic diameters of lipid-DOPE co-liposomes at optimized lipid/DOPE ratio and lipoplexes at optimized N/P ratio are shown in the Figure S2. We observed that the hydrodynamic diameters of the lipid-DOPE coliposomes were smaller than their lipoplexes except in the case of cationic lipid CholHG-3ox.

**Table 1:** Average hydrodynamic diameters and sizes of the lipid aggregates as obtained from the DLS measurements, and TEM studies respectively.

Lipid	Hydrodynamic Diameter (nm)^a^	Size from TEM (nm)^b^	Lipid Bilayer Width (Å)^c^	Zeta Potential (mV)
**CholG-D**	147±12	30–70	46.6	40±4
**CholHG-D**	212±13	40–110	46.1	65±1
**CholHG-1ox**	137±3	30–60	46.7	62±3
**CholHG-2ox**	154±4	30–90	47.8	57±1
**CholHG-3ox**	220±8	40–130	46.7	58±1
**CholHG-4ox**	149±2	40–60	42.2	41±0.5

The bilayer widths of the aggregates of the cationic gemini lipids as revealed from the x-ray diffractions.

a Hydrodynamic diameters as obtained from DLS measurements; each value is shown as the mean ± S.D. (standard deviation) (*n = *3).

b As evidenced from TEM.

c Lipid bilayer width from the XRD experiments; the error in the measurements of width were within ±1%.

#### Zeta potential measurements

Zeta potential (Table 1) values of these cholesterol-based cationic lipid aggregates in aqueous media ranged from 40–65 mV. Among the geminis having oxyethylene spacer as suspensions in water, CholHG-1ox has higher zeta potential (∼62 mV) whereas CholHG-4ox has lower zeta potential (∼41 mV). Among the lipids with OH function at the spacer, we observed that the CholHG-D aggregates (possessing -OH both on the headgroup and on the spacer) have higher zeta potential (∼65 mV) whereas that of the CholG-D (possessing -OH only on the spacer) have lower zeta potential (∼40 mV). Thus hydration effects on such gemini aggregates depended both on their nature and location.

#### Lipoplex formation as followed by zeta potential titrations

Zeta potential of aqueous solution of plasmid DNA pEGFP-C3 (4 µg/mL) was recorded as −8 mV which increased on the addition of either cationic gemini lipid CholHG-1ox (Figure S3A) or CholHG-3ox suspension (Figure S3B). CholHG-1ox (0.5 mg/mL) was able to make a lipoplex of maximum zeta potential of ∼12 mV at a N/P charge ratio of 1 whereas CholHG-3ox could get a maximum zeta potential of ∼9 mV only at N/P charge ratio 0.5. Inclusion of DOPE in cationic liposomes changed the potential of the lipoplexes further. Thus CholHG-1ox gave a lipoplex of ∼ 22 mV at N/P of 2 compared to CholHG-3ox lipoplex of ∼11 mV at N/P of 1.

Addition of fetal bovine serum (FBS) in water medium (10%) changed the pattern significantly. CholHG-1ox gave a lipoplex of maximum zeta potential of ∼18 mV at N/P of 0.5 compared to that of CholHG-3ox based lipoplex which gave a zeta potential of ∼12 mV at N/P of 0.25. The electro-neutrality of CholHG-1ox was achieved between 0.125 and 0.25 which shifted to 0.5–0.75 on addition of DOPE while the presence of FBS made it between 0.125 and 0.25 again. The electroneutrality of CholHG-3ox was achieved between 0.125 and 0.25 which decreased to 0–0.125 on addition of DOPE while the presence of FBS did not affect the electroneutrality N/P charge ratio.

#### X-ray Diffraction (XRD) studies

From the XRD studies (Table 1), we observed that among the geminis with oxyethylene spacer, CholHG-2ox has the highest lipid bilayer width (∼48 Å) whereas CholHG-4ox has the lowest lipid bilayer width (∼42 Å). For the lipids with hydroxyl function on the spacer, the CholHG-D and CholG-D have almost comparable bilayer widths (∼46 Å).

#### Effect of DOPE and FBS on DNA Complexation by Lipid Aggregates

**Figure 4:**
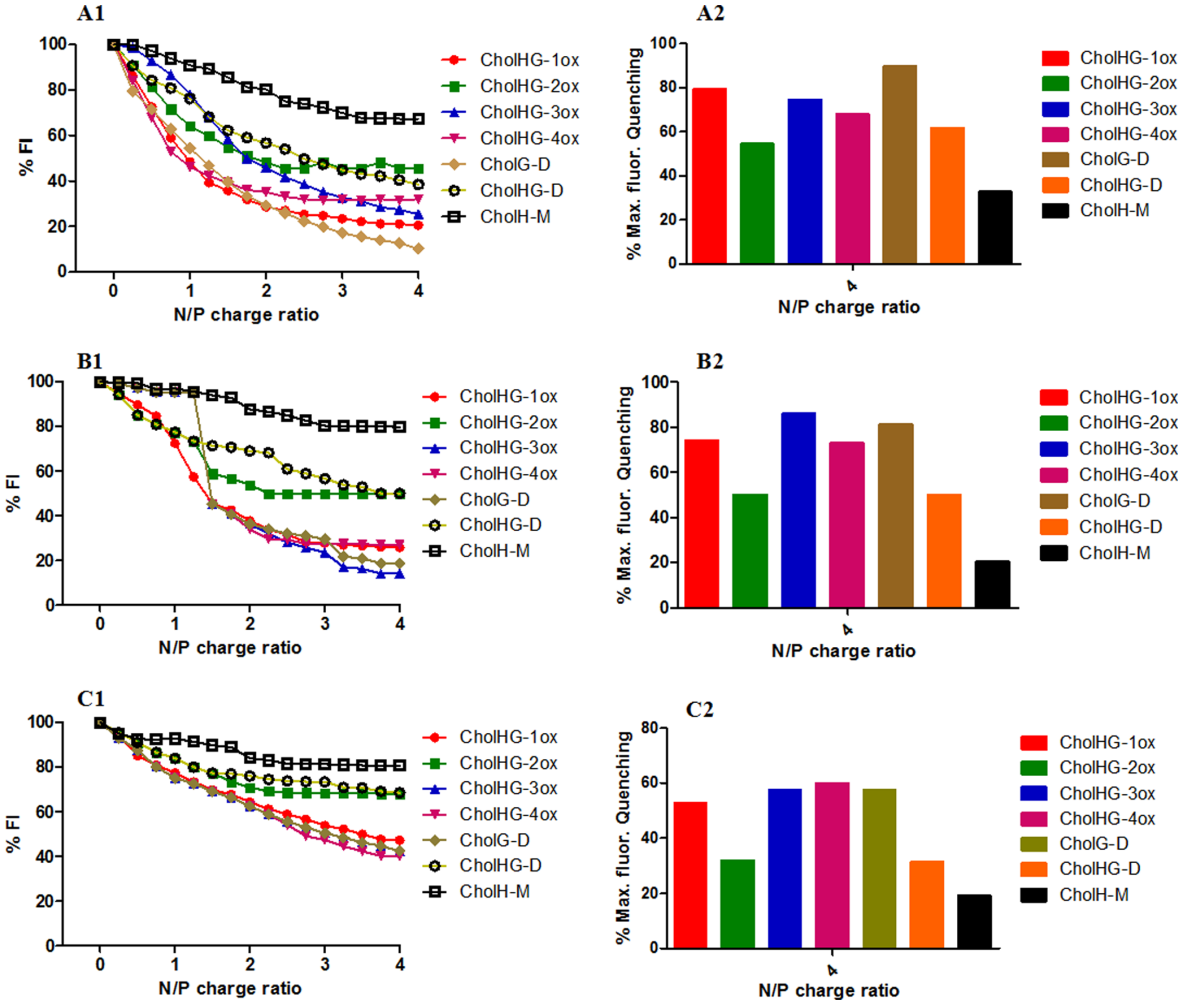
Ethidium bromide exclusion assay. Release of ethidium bromide (EB) from DNA–EB complexes upon addition of each cationic cholesterol gemini lipid suspension at different lipid/DNA charge ratios. Experiments were performed using liposomes possessing (A1–A2) cationic lipids, (B1–B2) cationic lipid:DOPE (1∶1; molar ratio) and (C1–C2) cationic lipid:DOPE (1∶1; molar ratio) in presence of 10% FBS serum. Graph A1, B1 and C1 represent gradual decrease in % FI of EB due to EB exclusion across N/P charge ratios 0–4 whereas histogram A2, B2 and C2 represent % max. fluorescence quenching at N/P charge ratio 4.

Upon intercalation of EB (7 µM) into the plasmid-DNA (50 µM), a fluorescence emission with a λmax ∼592 nm was obtained. When a given cationic lipid suspension (0.8 mM) was added incrementally into the EB/plasmid DNA solution, a gradual quenching of the EB fluorescence emission was observed which eventually led to saturation as shown in Figure 4A1 and A2 [39].

From the EB exclusion assay, we found that the cationic gemini cholesterol aggregates induced the release of EB from the EB/DNA complexes in the range of 55–90%. Among all the geminis, CholH-M was the least efficient in DNA binding than its gemini counterparts. Within the geminis with oxyethylene spacer, the liposomes of CholHG-1ox facilitated the dissociation of EB from EB-DNA complex to an extent of ∼80% at a lipid:DNA ratio of 4.0 whereas the liposomes of CholHG-2ox showed the lowest EB exclusion (∼55%). For the lipids with hydroxyl group on the spacer, we observed that the liposomes of CholG-D (having hydroxyl functionality only on the spacer) facilitated the dissociation of EB from EB-DNA complex to an extent of ∼90% at a lipid:DNA ratio of 4.0 whereas the CholHG-D liposomes (having OH function both on the headgroup and on the spacer) showed lower EB exclusion (∼70%) from the EB-DNA complex. Thus the presence of OH group both on the headgroup and on the spacer influenced the efficiency of lipoplex formation with DNA.

Further, we investigated the effect of DOPE inclusion on the DNA binding efficiency of cationic cholesterol lipids by performing the EB exclusion assay as mentioned above by using 1∶1 mole ratio liposome of lipid:DOPE (Figure 4B1 and B2). It was found that in all the cases the DNA binding efficiency decreased by 5–10% whereas CholH-M showed maximum decrease of ∼10%.

We also investigated the effect of FBS (10%) on the DNA binding efficiency of the cationic cholesterol lipids (Figure 4C1 and C2). Experiment was performed as mentioned above using lipid:DOPE formulations. Fluorescence data were recorded in presence of 10% FBS in sample possessing EB, DNA and lipid:DOPE liposomes. It was observed that the presence of FBS further decreased the DNA binding efficiency to a considerable extent. In presence of 10% serum, where CholHG-1ox could bind only ∼60% of DNA at N/P charge ratio of 4, the monomeric lipid CholH-M could bind only as little as 15%. Probably FBS exerts specific affinity towards these cationic cholesterol lipids.

#### Effect of DOPE and FBS on the SDS-Induced release of DNA from the lipoplexes

Negatively charged micellar solution of SDS is known to induce release of DNA from various lipoplexes [40]. Such anionic micelles mimic the negatively charged phospholipids present in endosomes. Recently Cardoso *et al.* has shown that transfection-competent formulations can be efficiently destabilized by interaction with different anionic and zwitterionic bilayers, including those containing phosphatidylserine and cardiolipin [41].

**Figure 5:**
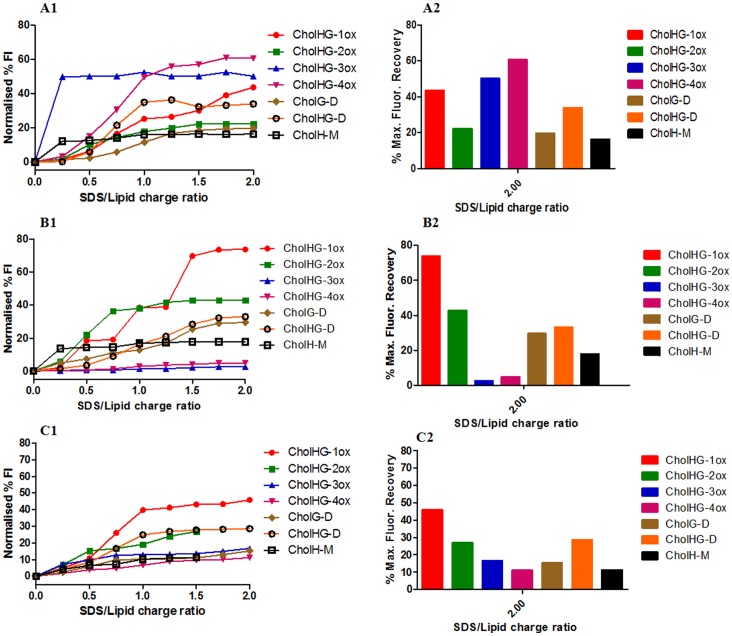
Ethidium bromide re-intercalation assay. Re-intercalation of ethidium bromide (EB) to DNA released from each lipoplex upon addition of miceller SDS at different SDS/lipid charge ratios for cholesterol-based cationic gemini lipid. Experiments were performed using liposomes possessing (A1–A2) cationic lipids, (B1–B2) cationic lipid:DOPE (1∶1; molar ratio) and (C1–C2) cationic lipid:DOPE (1∶1; molar ratio) in presence of 10% FBS serum. Graph A1, B1 and C1 represent gradual increase in % FI of EB due to EB exclusion across SDS/lipid charge ratios 0–2 whereas histogram A2, B2 and C2 represent % max. fluorescence recovery at SDS/lipid charge ratio 2.

Among all the lipids, the liposomes of CholHG-4ox were most efficient in facilitating the DNA release (60%) from the lipoplexes in presence of negatively charged micelles at a maximum SDS: lipid molar ratio of 2. Among the geminis with oxyethylene spacer, the liposomes of CholHG-2ox were the least effective (upto 20%) in releasing DNA from the lipoplexes at a maximum SDS: lipid ratio of 2 but CholH-M was the least efficient among all lipids. For the lipids with hydroxyl functionality located on the spacer chain, the liposomes of CholHG-D facilitated the dissociation of DNA from the corresponding lipoplexes better than the CholG-D at SDS: lipid ratio of 2. Thus at fixed SDS/lipid charge ratio of 2, the release of DNA from the lipoplexes followed the order: CholHG-4ox>CholHG-3ox>CholHG-1ox>CholHG-D>CholHG-2ox>CholG-D>CholH-M for the cholesterol-based cationic lipids (Figure 5A1, A2).

Further, we investigated the effect of DOPE inclusion in the cationic liposomes on SDS-induced release of DNA from the lipoplexes. Experiment was performed using 1∶1 molar ratio of lipid:DOPE (Figure 5B1 and B2). It was found that in all the cases DNA release efficiency was drastically reduced in the case of all the lipids except CholHG-1ox in presence of DOPE. CholHG-1ox showed an increase in DNA release efficiency compared to the liposomes devoid of DOPE whereas CholHG-3ox and Chol-4ox showed only ∼15% DNA release.

We also investigated the effect of FBS (10%) on DNA release efficiency of cationic cholesterol lipids (Figure 5C1 and C2). Here fluorescence data were recorded in presence of 10% FBS in samples possessing EB, DNA and lipid:DOPE suspensions and SDS. It was observed that presence of FBS further decreased the DNA release efficiency to a considerable extent. In presence of 10% serum, CholHG-1ox released ∼50% DNA at SDS: lipid molar ratio of 2. Other lipids could release a maximum of 30% of bound DNA under the analogous conditions.

#### DNA binding and release assay

**Figure 6:**
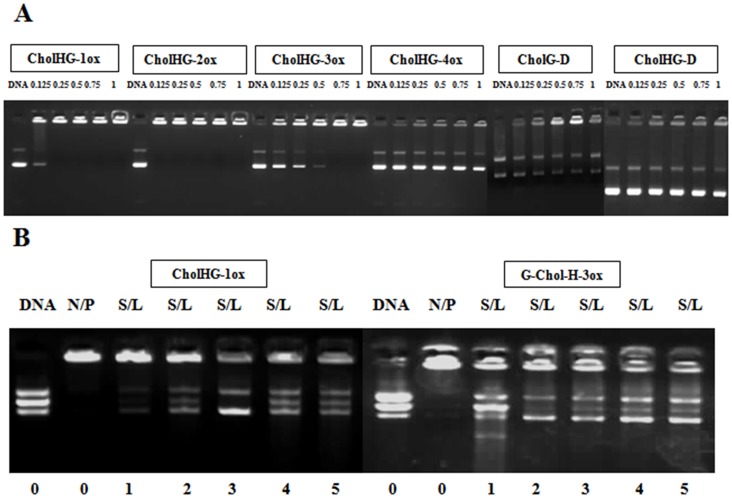
Gel electrophoresis to find out DNA binding and release efficiency. Electrophoretic gel patterns for the lipoplex-associated pEGFP-C3 plasmid DNA. (A) DNA binding efficiency of different gemini lipid based lipoplexes. The N/P ratios are indicated at the top of each lane. (B) SDS mediated DNA release from representative lipid based lipoplexes. The SDS/lipid ratios are indicated below each lane. Both experiments were performed using 0.2 µg of DNA per well.

Each cationic lipid suspension was able to retard plasmid DNA inside the well at particular N/P ratio (Figure 6). The binding efficiency decreased with an increase in the spacer length in the following order of CholHG-2ox>CholHG-1ox>CholHG-3ox>CholHG-4ox>CholG-D>CholHG-D. Release of DNA from the lipoplexes was examined with two representative formulations CholHG-1ox and CholHG-3ox. CholHG-3ox showed better DNA release ability than that of CholHG-1ox.

### C. Transfection Biology

#### Formation of mixed liposomes with DOPE

Liposomes could be conveniently prepared from each gemini lipid with naturally occurring helper lipid DOPE by first subjecting the films of lipid mixtures to sufficient hydration, repeated freeze-thaw cycles followed by sonication at 70°C for 15 min.

#### Optimization of Lipid:DOPE ratio

Experiments were performed in the absence (Figure S4) and in the presence of serum (Figure S5). Each gemini lipid was most effective at the lipid:DOPE mole ratio of 1∶1, except lipids CholG-D and CholHG-D which were most effective at lipid: DOPE mole ratios of ∼1∶2 in absence of serum (−FBS−FBS). In presence of serum (−FBS+FBS), the N/P charge ratio for optimized transfection efficiency increased from 1∶1 to 1∶4 while it was found as 1∶2 for both CholG-D and CholHG-D based formulations. To our pleasant surprise, the mean fluorescence intensity (MFI) values were better in presence of serum compared to those without serum. Probably the spacer type and length play important role in the generation of optimal lipid: DOPE formulations, as the amount of DOPE decreased from 4-fold to equimolar ratio with the increase in the spacer lengths from CholHG-1ox to CholHG-4ox. Probably these lipids with oxyethylene spacer interact with the FBS ingredients (i.e., its anionic proteins) in some fashion that might render the DNA-lipid complexes “loose” at higher lipid/DOPE ratio. Presence of zwitterionic DOPE optimizes surface fusogenicity in favor of high efficiency [42]. In absence of serum, lipoplexes do not face the above problem and only minimum molar ratio suffices (1∶1 for CholHG-1ox, CholHG-2ox and CholHG-3ox while 1∶2 for CholHG-4ox, CholG-D and CholHG-D). In case of CholG-D and CholHG-D, presence of FBS however, did not make any difference in the optimization ratio with DOPE and remained constant at 1∶2.

CholHG-1ox, CholHG-2ox, CholHG-3ox and CholHG-4ox acted as better transfecting agents compared to CholG-D and CholHG-D in absence of serum whereas in serum CholHG-1ox was found to be the best formulation and CholHG-2ox, CholHG-3ox and CholHG-4ox were also better than that of CholG-D and CholHG-D.

#### Optimization of N/P ratio

Experiment was performed with N/P variation from 0.125 to 3. In absence of serum, CholHG-1ox was able to transfect to the maximum extent of ∼70% of the cells with MFI of ∼35 at N/P ratio of 3, whereas CholHG-2ox could transfect approximately ∼85% of the cells with nearly identical MFI at the same N/P ratio (Figure S6). CholHG-3ox was able to transfect ∼80% of cells at N/P of 2 with nearly same MFI observed as in case of CholHG-1ox and CholH-2ox. Similarly formulations based on each one of CholHG-4ox, CholG-D and CholHG-D transfected ∼80% of the cells with MFI of ∼35 at N/P ratio of ∼1.

**Figure 7:**
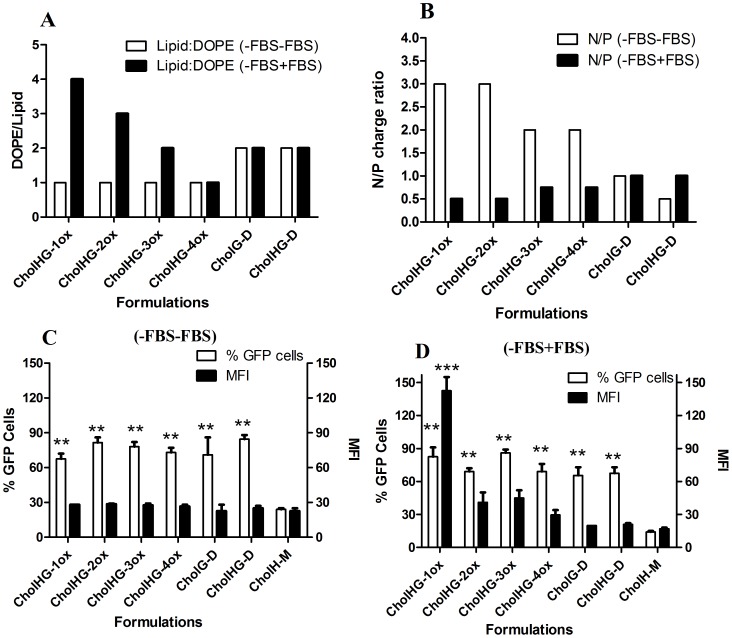
Optimized formulations of different cholesterol based lipids. (A) Optimized DOPE:lipid ratios; (B) optimized N/P ratios; (C) best transfection efficiency of lipids in absence of serum and (D) best transfection efficiency of lipids in serum. Concentration of DNA = 0.8 µg/well. Data are expressed as number of transfected cells and MFI as obtained from flow cytometry. Statistical differences from the controls (CholH-M) are labelled ** *P<*0.005 and *** *P<*0.0005.

In presence of serum, CholHG-1ox was however, able to transfect more efficiently (to maximum extent of ∼90% of the cells) with higher MFI of ∼150 at N/P ratio of 0.5, whereas CholHG-2ox could transfect ∼70% of the cells with a MFI of ∼50 at the same N/P ratio (Figure S7). Transfection efficiency decreased at higher N/P ratio. CholHG-3ox was able to transfect ∼90% of cells at N/P of 0.75 with nearly same MFI observed as in case of CholHG-2ox. Similarly, CholHG-4ox, CholG-D and CholHG-D were able to transfect maximum extent of ∼70% of the cells with MFI of ∼30 at the N/P ratio of ∼1. When all the geminis were compared at their optimized N/P ratios, CholHG-1ox was found to be the best transfecting agent in terms of MFI, although the number of transfected cells mediated by CholHG-1ox was found be comparable to other gemini lipids. Thus the transfection efficiency decreased with the increase in the spacer lengths from CholHG-1ox to CholHG-4ox and while going from CholG-D to CholHG-D, there was no significant change (Figure 7). Each gemini lipid was invariably found to be significantly better transfecting agents compared to the monomeric species (** p<0.005) in both absence (−FBS−FBS) and presence of serum (−FBS+FBS).

All the negative controls *viz.* cells treated with pEGFP-C3 alone, CholHG-1ox, CholHG-3ox, lipoplex CholHG-1ox/PGL3 and lipoplex CholHG-3ox/PGL3 were analyzed along with CholHG-1ox/pEGFP-C3 and CholHG-3ox/pEGFP-C3 with FACS for quantitation of GFP where PGL-3 was a non-GFP expressing plasmid (Figure S8). Data showed that all the negative controls gave a fluorescence intensity much lower than CholHG-1ox/pEGFP-C3 and CholHG-3ox/pEGFP-C3 and did not give any false positive value for % GFP cells [43], [44]. Lipoplexes with PGL-3 were prepared using the same optimized N/P charge ratio as used in case of pEGFP-C3 plasmid. Experiment was performed in 10% serum condition.

#### Effect of the amount of DNA

To see how a variation in the amount of DNA affects transfection efficiency of gemini lipids, we performed transfection with best gemini lipid at fixed N/P ratio of 0.5 and at pre-optimized DOPE: lipid molar ratio of 4∶1, varying the amount of the DNA from 0.4 to 2.0 µg/well (Figure S9). In case of CholHG-1ox, 0.8 µg of DNA was found to be the best under our standardized conditions. Any variation from this amount of DNA decreased the % of transfected cells and in MFI.

#### Effect of serum on transfection efficiency

**Figure 8:**
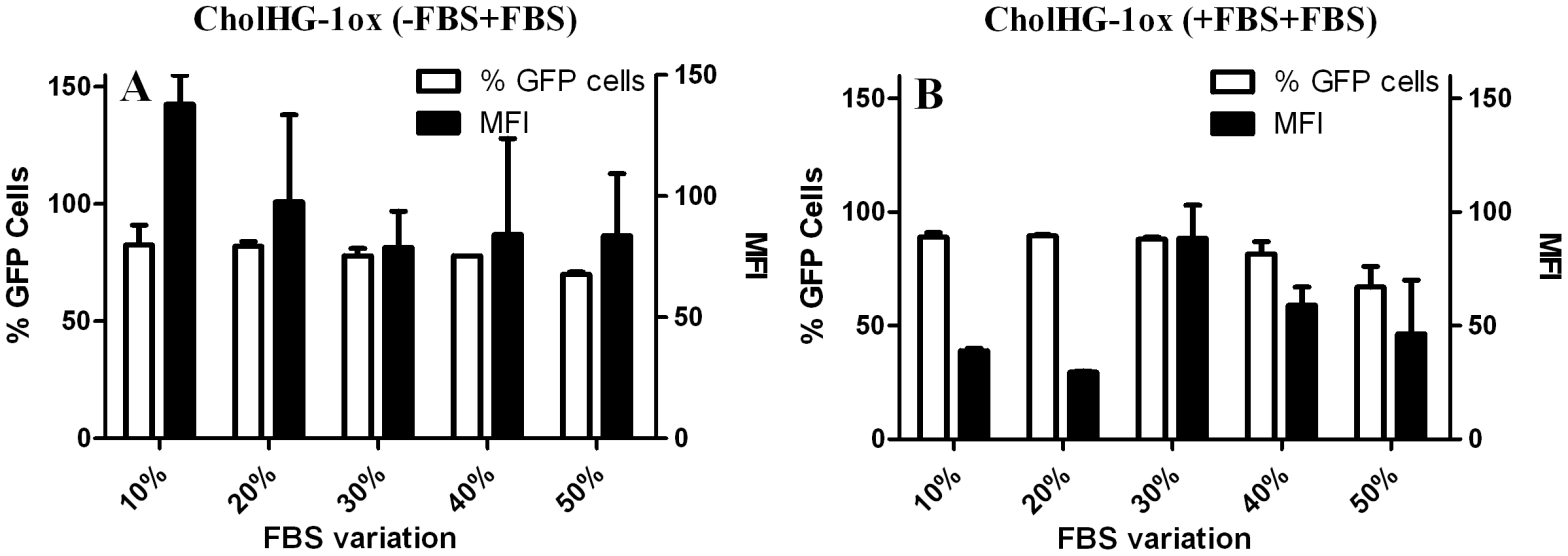
Effect of increasing FBS concentrations on gene transfection efficiency of CholHG-1ox: DOPE (1∶1). Concentration of the DNA = 0.8 µg/well. Data are expressed as number of transfected cells and MFI as obtained from the flow cytometry. Transfections were performed (A) when lipoplex was prepared in absence of serum but incubated with cells in presence of serum (−FBS+FBS) and (B) when lipoplex was prepared as well as incubated with cells in presence of serum (+FBS+FBS).

In order to investigate the effect of high serum percentages on the gene transfection efficiencies of cholesterol based lipids, we performed transfection in presence of serum with optimized lipid: DOPE formulation at different N/P ratios using plasmid pEGFP-C3. The results were analyzed by flow cytometry (Figure 8). Interestingly a significant increase in the transfection efficiency of the lipid CholHG-1ox was observed in presence of 10% serum as compared to the one carried out without serum (Figure 7C and 7D). CholHG-1ox: DOPE (1∶1) based formulation was able to transfect only ∼70% of the cells with a MFI of ∼30 without serum. However, in presence of 10% serum, the transfection efficiency increased to 90–95% with a MFI of ∼160 at optimized N/P ratio. This suggests that some serum components probably facilitate the transfection activity with liposomes prepared from CholHG-1ox. Such an increase in the transfection efficiency occurred with other geminis as well although significant increase was observed in terms of % GFP cells only.

Probably, the presence of cholesterol moiety in the molecular structures of the presented geminis, increases the stability and transfection efficiency of such lipoplexes in serum. Indeed it was reported recently by Betker *et al.* that the cholesterol domain formation significantly improves the transfection by serum protein binding in certain formulations [45]. Further, the difference in transfection efficiency in presence of serum among all the geminis could be explained on the basis of their biophysical characteristics. It was found that CholHG-1ox improved the lipoplex formation during zeta potential measurements in presence of 10% serum while CholHG-3ox remained unaffected. It was also found that CholHG-1ox released ∼50% DNA at SDS: cationic lipid molar ratio of 2, whereas other lipids could release a maximum of only ∼30% of the lipoplex bound DNA under the analogous condition of 10% serum environment. Probably, thus the better responsiveness of CholHG-1ox toward plasmid DNA in presence of serum makes it a better transfecting agent compared to the other geminis in serum.

We then wanted to find out the effect of even higher serum concentrations on transfection efficiency. Toward this end, experiments were performed in two different conditions. First lipoplexes were prepared in absence of serum and incubated with cells in presence of serum (−FBS+FBS). In other case lipoplexes were both prepared and incubated with the cells in presence of serum (+FBS+FBS). Both experiments were performed where the percentages of serum were varied from 10 to 50% (Figure 8). Surprisingly, even in very high serum concentration (50%), formulation based on CholHG-1ox was able to transfect ∼70% cells with a MFI of ∼80 (Figure 8A) while in other case ∼70% cells were GFP positive with MFI of ∼60 (Figure 8B). These findings are significant in that it was possible to optimize a lipid formulation for such a level of transfection efficiency even at a very high serum concentration, while lipoplex was also prepared in serum. When experiment was performed in –FBS+FBS condition, with increase of FBS concentration, not much decrease in % GFP cells was observed although MFI decreased progressively with increase in FBS from 10 to 50%. FBS is known to destabilize lipoplexes [46] and this in turn decreases transfection efficiency. However, when experiment was performed in +FBS+FBS condition, there was not much decrease in % GFP cells transfected although MFI showed a ‘bell’-shaped serum concentration dependence behavior when FBS percentage was increased from 10 to 50%. With 30% FBS, CholHG-1ox showed a maximum MFI value of ∼95 while at 10 and 20% FBS, the MFI was merely ∼50 and ∼40 respectively and in 40 and 50% FBS condition, the CholHG-1ox formulation transfected cells with a maximum MFI of ∼70 and ∼60 respectively. Probably, in –FBS+FBS condition, the lipoplexes were destabilized in presence of anionic proteins present in FBS and this in turn caused a decrease in the transfection efficiency, especially in terms of MFI. In case of +FBS+FBS, the lipoplex was formed from a mixture of plasmid DNA and CholHG-1ox in presence of anionic proteins of FBS, which afforded new equilibrium compositions of DNA, anionic proteins and lipid with increasing amount of FBS.

Lipid CholHG-1ox was found to be a better transfecting agent even in transformed human embryo kidney (HEK 293T) cells compared to Effectene in 50% serum (Figure S10A and S10B). It was found considerably biocompatible at all the concentrations and N/P charge ratios (Figure S10C).

#### Comparison of transfection efficiency

Optimal transfection efficiencies of each gemini lipid were compared with that of the corresponding monomeric lipid (CholH-M) both without (Figure 7C) and with serum (Figure 7D). Each gemini was found to be better transfecting agent in serum. In presence of serum, their efficiency was enhanced further in terms of both % GFP cells as well as the MFI relative to the monomer. Overall the gemini CholHG-1ox was found to be the best transfecting agent in the series with ∼90% GFP and 150 MFI in 10% serum.

**Figure 9:**
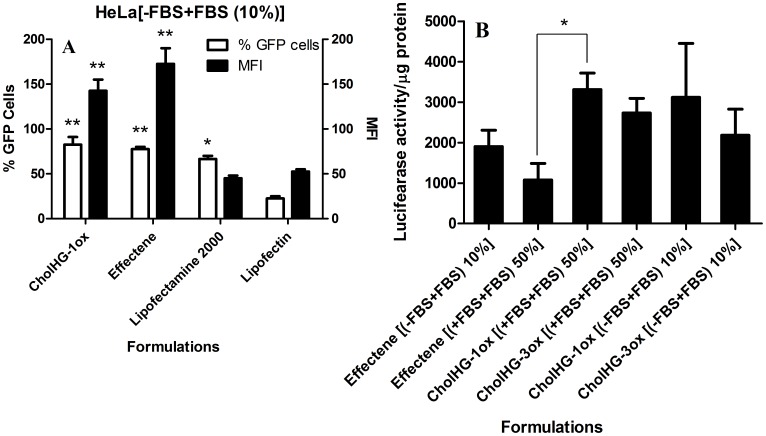
Transfection efficiency of the gemini (CholHG-1ox) based formulation against various commercial transfection reagents in presence of serum. Concentration of DNA = 0.8 µg/well. (A) Data are expressed as number of transfected cells and MFI as obtained from flow cytometry analysis. (B) Data are expressed as luciferase activity/µg of protein, extracted from transfected cells. Statistical differences from the controls (Lipofectin in 8A) are labeled * *P<*0.05 and ** *P<*0.005.

Transfection efficiency of the best formulation CholHG-1ox here was then compared with three different, commercially available transfection reagents, e.g., Lipofectin, Lipofectamine 2000 and Effectene in presence of serum (Figure 9A). CholHG-1ox was again found to be the best transfecting agent in terms of % GFP cells while Effectene was found to be better in terms of MFI, when transfection was performed in 10% FBS and the DNA-transfecting agent complexes were prepared without serum (−FBS+FBS). To independently quantify transfection efficiency in serum, we also examined transfection mediated by above reagents and gemini based formulations using LAR II reagent (Promega) based luciferase gene expression. In (+FBS+FBS) conditions, when lipoplexes or transfection reagent-DNA complexes were prepared as well as incubated with cells in serum at very high serum (50%), CholHG-1ox was found to be even better than that of Effectene (Figure 9B). These results were compared with the transfection efficiencies at 10% serum (−FBS+FBS) for CholHG-1ox, Effectene as well as CholHG-3ox. CholHG-1ox was found to be >3 times better transfecting agent at 50% serum conditions (+FBS+FBS) compared to Effectene. Even CholHG-3ox was found to be better than Effectene in presence of 50% serum, which is one of the relatively less effective transfecting agents in this series of gemini lipids.

#### Fate of DNA in lipoplexes in serum

**Figure 10:**
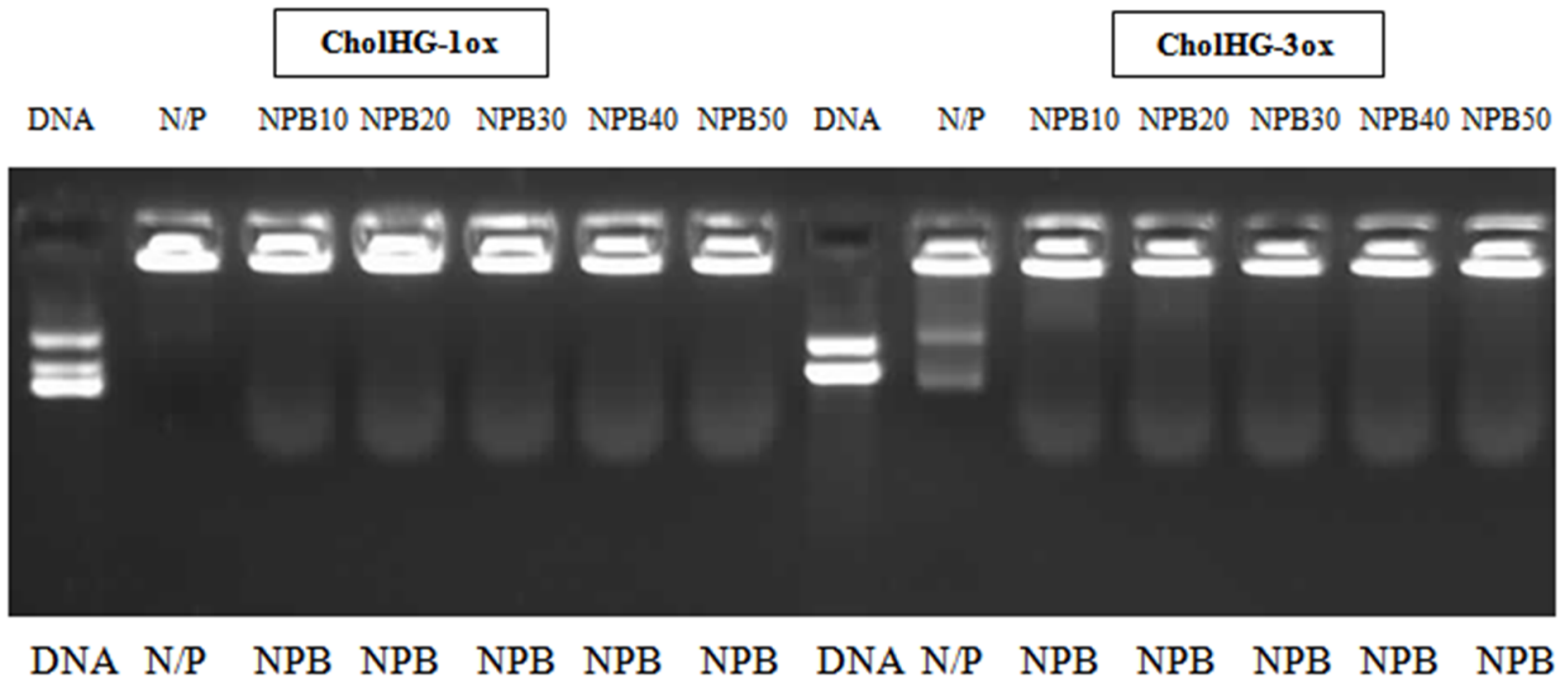
Bovine serum albumin (BSA) induced gel retardation. Gel electrophoretic patterns for the lipoplex-associated pEGFP-C3 plasmid DNA in the gel retardation assay for cationic lipid formulations where complexes were further treated with BSA. Experiment was performed using 0.2 µg of DNA per well at the N/P ratio of 5.

Blood serum, which consists of negatively charged proteins, is known to dissociate DNA from its lipoplexes due to a competition with DNA for cationic lipid molecules [47]. Indeed serum decreases the lipoplex stability during gene delivery and affects overall reporter gene expression [47]. However, surprisingly, in the present instance with gemini CholHG-1ox based formulations, blood serum was found to be a stabilizing factor. Here we used two formulations based on geminis CholH-1ox and CholHG-3ox which represented a good and an average transfection agent respectively (Figure 10). A N/P ratio of 0.5 was chosen for the lipoplex formation. Under this condition, both geminis could retard ∼90% of the added DNA to the wells. But on addition of 10% FBS to pre-formed lipoplexes at N/P 0.5, the resulting mixtures were totally confined to the wells indicating no dissociation of DNA from its lipoplexes in serum. Probably, the presence of -OH moiety on the headgroups of both geminis, might be responsible for an enhanced lipoplex association with serum without leading to any dissociation of DNA from the resulting serum bound lipoplexes.

#### DNase I stability of lipid-DNA formulations

Nucleases are responsible for the degradation of DNA in cytosolic environments. Thus, DNA stability of a given lipoplex formulation in presence of enzymes such as DNase I is an important indicator that predicts its transfection efficiency (Figure S11). Here we used two geminis, CholHG-1ox and CholHG-3ox based liposomes as before and an N/P ratio of 1 was selected for the lipoplex formation. Pre-formed lipoplexes without any FBS and in presence of 10% FBS as well as equivalent amount of BSA were incubated with DNase I for different time periods and finally the reaction mixtures were analyzed upon electrophoretically running on 1% agarose gel. In this experiment, 0.25 unit of DNase I was used for incubation and the incubation period was varied from 2, 4 to 6 h. Experiment showed an increase in lipoplex stability with FBS as well as BSA. It further showed an improved stability of DNA in CholHG-1ox derived lipoplexes in presence of FBS or BSA against DNase I. Probably, due to better resistance of CholHG-1ox-DNA lipoplexes to DNase in presence of serum, the transfection efficiency of CholHG-1ox was significantly better than that of CholHG-3ox. Higher stability of DNA in lipoplexes prepared from CholHG-1ox in presence of BSA or FBS explains its higher transfection efficiency compared to CholHG-3ox in serum.

#### Cytotoxicity assay

**Figure 11:**
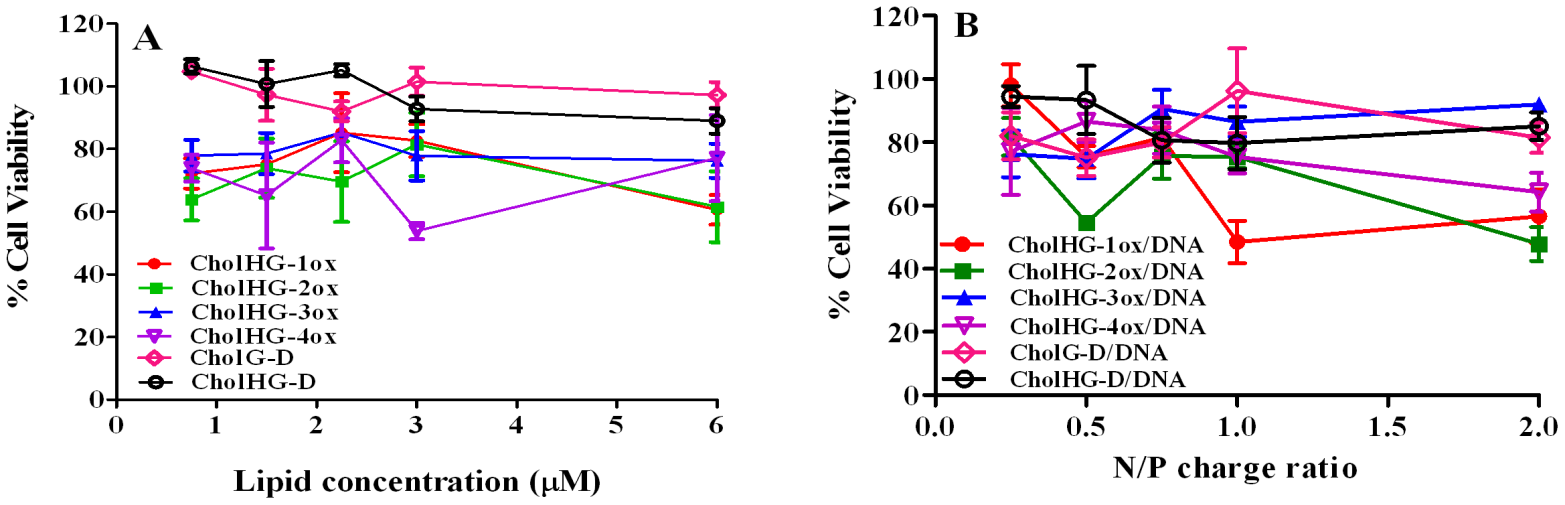
MTT based cellular cytotoxicity assay of the lipoplexes at different N/P ratios. Experiment was performed using optimized lipid: DOPE and pEGFP-C3 plasmid DNA against HeLa cells. The percentage viability values shown are the average of triplicate experiments performed on the same day. (A) Cytotoxicity of lipids at different concentrations varying from 0.6 to 3 µM. Same concentrations were used for transfection during the lipoplex preparation. (B) Cytotoxicity of lipoplexes at different N/P charge ratios, which were used during transfection.

MTT-based cell viability assays were performed in HeLa cells across the entire range of lipid: DNA charge ratios (N/P) as well as concentration of corresponding gemini lipids present in lipoplexes used in the actual transfection experiments. Cell viabilities in presence of each gemini formulations except CholHG-1ox and CholHG-2ox were found to be high at all the concentrations (Figure 11; Figure S12A, S12B). CholHG-1ox and CholHG-2ox were found to be slightly toxic at higher concentrations (50 µM).

Cytotoxicity assay for commercial reagent “Effectene” was performed in HeLa cells, which were grown in 96-well plates for 24 h prior to the treatment. Experiments were performed in presence of 10% serum condition using the Effectene/DNA ratios used for other transfection experiments (Figure S12C). Effectene was found slightly more toxic to the cells compared to our formulations, alone or along with plasmid DNA as it showed only ∼75% cells were viable.

#### BrdU incorporation assay

The results of the cell proliferation assay experiments [48], [49] with various liposomes and lipoplexes in HeLa cells are shown in Figure S13. All the liposomes and lipoplexes have no significant effect on the inhibition of DNA synthesis and cell proliferation in the presence of –FBS+FBS (10%). Only a mild inhibitory effect was observed in +FBS+FBS (50%) condition. Thus, high transfection efficiency in case of high serum percentage is surely not due to high cell viability but due to better lipoplex stability in presence of lipid, DOPE and FBS.

#### Transfection efficiency by fluorescence microscopy

**Figure 12:**
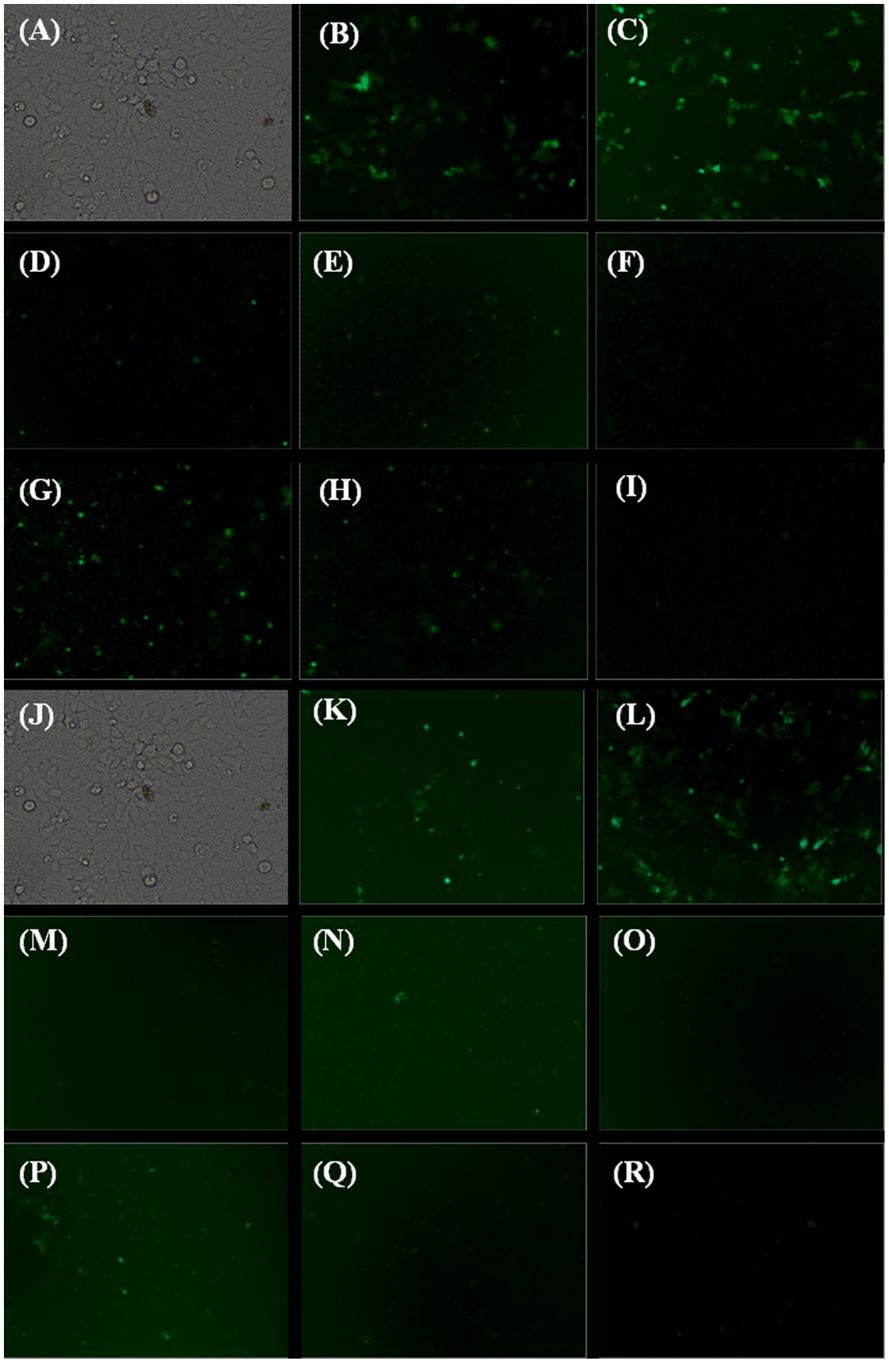
Fluorescence microscopic imaging of pEGFP-C3 transfected HeLa cells. Cells were transfected of 50% FBS: (A–I) −FBS+FBS and (J–R) +FBS+FBS. Cells were treated with (A and J) Cells only; (B and K) Effectene using manufacturers protocol; (C and L) CholHG-1ox, N/P ratio 0.5; (D and M) CholHG-2ox, N/P ratio 0.5; (E and N) CholHG-3ox, N/P ratio 0.75; (F and O) CholHG-4ox, N/P ratio 0.75; (G and P) CholG-D, N/P ratio 1; (H and Q) CholHG-D, N/P ratio 1 and (I and R) Chol-M, N/P ratio 4. Plasmid DNA pEGFP-C3, 0.8 µg was used in study.

Green fluorescence protein expression was observed under fluorescence microscope at the end of 48h of post-transfection incubation. CholHG-1ox expressed higher amount of GFP (Figure 12C,L) compared to all other lipids including Effectene (Figure 12B,K) in both –FBS+FBS and +FBS+FBS (50%) condition. Further, fluorescence in each instance was quantified using FACS (Figure S14). CholHG-1ox was able to transfect ∼3 fold more cells compared to Effectene in both the conditions (Figure S14A). The corresponding histogram also showed better MFI with this lipid (Figure S14B,C). Overall, CholHG-1ox was found to be significantly better transfecting agent compared to commercially available Effectene.

#### Confocal studies

Confocal studies were performed on HeLa cells using gemini formulations CholHG-1ox and CholHG-3ox in absence and presence of serum along with commercial reagent Effectene (Figure S15). Each gemini formulation showed the presence of GFP in the cytosolic region of cells and was comparable or better than that delivered by Effectene. HeLa cells transfected with CholHG-1ox: DOPE (1∶1) in presence of serum (−FBS+FBS) express more GFP (Figure S15B) compared to those transfected with CholHG-1ox: DOPE (1∶1) in absence of serum (−FBS−FBS) (Figure S15A). This observation is consistent with our finding of FBS mediated enhancement in transfection efficiency of this series of transfecting agents. Even the gemini formulations are better than Effectene (1∶25) both in absence of serum (−FBS−FBS) (Figure S15C) and in presence of serum (−FBS+FBS) (Figure S15D) in terms of the expression of GFP. In 10% serum (−FBS+FBS) CholHG-1ox: DOPE (1∶1) expressed more GFP (Figure S15E) compared to HeLa cells transfected with CholHG-3ox: DOPE (1∶1) (Figure S15F). Similarly in 50% serum (−FBS+FBS), CholHG-1ox: DOPE (1∶1) expressed more GFP (Figure S15G) than CholHG-3ox: DOPE (Figure S15H). Taken together this indicates that serum supports CholHG-1ox more than CholHG-3ox in terms of transfection efficiency.

### Conclusions

For the first time, four gemini cationic cholesterols with varying lengths of oligo-oxyethylene based spacer chains were synthesized, that also possessed -CH_2_CH_2_OH groups at their headgroups. Two additional cholesterol based geminis were also synthesized that contain hydroxyl groups on their spacer segments which connect two cationic ammonium groups, one of which also have -CH_2_CH_2_OH group. Each lipid formed stable suspensions in water which were confirmed to be vesicles by TEM. CholHG-3ox formed the largest aggregates whereas CholHG-1ox formed the smallest particles based on DLS studies. Aggregates of CholHG-D showed the highest zeta potential (∼65 mV).

Variations observed in the zeta potential values of the cationic gemini lipids indicate their interaction with FBS. Pronounced changes in the zeta potential with CholHG-1ox compared to CholHG-3ox probably indicate greaterinteraction between the anionic proteins of FBS and CholHG-1ox. The difference in the gemini lipid headgroups and spacers may be responsible for the difference in interaction between a given gemini lipid and FBS proteins. EB exclusion assay has shown that liposomes of CholHG-1ox facilitate the dissociation of EB from the EB-DNA complex to an extent of ∼94% indicating their strong propensity toward DNA based lipoplex formation. Release of DNA from different lipoplexes showed that CholHG-1ox based aggregates were more efficient than others in facilitating the release of DNA from their lipoplexes in presence of negatively charged micelles.

These gemini lipids in presence of helper lipid, DOPE showed a significant enhancement in the gene transfection activities as compared to their monomeric lipid counterpart CholH-M under comparable conditions. Co-liposomes of each gemini lipid with DOPE were not toxic at the concentrations at which transfections were performed. All gemini lipids except monomeric lipid showed either enhanced or sustained the same level of transfection activity in the presence of serum. All gemini lipid/DOPE formulations were able to show GFP expression both in absence and presence of serum as confirmed from the confocal images. With increase in the spacer from CholHG-1ox to CholHG-4ox, the transfection efficiency decreased whereas the presence of hydroxyethyl moiety at the spacer led to further decreases in the transfection activity.

Lipid CholHG-1ox was the most effective lipid in this series, which showed superior transfection activity than Effectene, even in 50% serum concentration. Interestingly, in presence of FBS and BSA, CholHG-1ox provided enhanced protection of plasmid DNA against DNase I. The protection was found to vary with the variation in the structural features of cholesterol based cytofectins. Hydroxyethyl moieties present on the headgroups in combination with oxyethylene type spacer thus provide an optimized combination of new genera of gemini lipids possessing high transfection efficiency even in presence of very high levels of serum (Figure S16). Despite the use of currently optimized lipofection conditions, including transfection in serum-depleted media, the efficiency of gene transfer is low and high transfection rates often induce cytotoxicity [50]. A lipid formulation with transfection efficiency not inhibited by serum would provide an advance towards possible *in vivo* applications.

## Materials and Methods

All reagents, solvents, and chemicals used in this study were of the highest purity available. The solvents were dried prior to use. Monomeric cholesterol based lipid CholH-M was synthesized as reported elsewhere [20]. Column chromatography was performed using 60–120 mesh silica gel. NMR spectra were recorded using a Jeol JNM λ-300 (300 MHz for ^1^H and 75 Hz for ^13^C) spectrometer. The chemical shifts (*δ*) are reported in ppm downfield from the internal standard, TMS, for ^1^H-NMR spectra. Mass spectra were recorded on a Kratos PCKompact SEQ V1.2.2 MALDI-TOF spectrometer, a MicroMass ESI-TOF spectrometer.

### General Method for Synthesis of Gemini Lipids

A solution of a particular amine either (cholest-5-en-3*β*-oxyethan-*N*-methyl-*N*-2-hydroxyethylamine or cholest-5-en-3*β*-oxyethan-*N*,*N*-dimethylamine) [20] (0.2 mmol) and either an appropriate α,*ω*-dibromoalkoxyalkane or 1,4-dibromobutane-2,3-diol (0.07 mmol) in dry MeOH-EtOAc (4 mL, v/v: 1/1) were mixed and then heated together at 80°C over a period of 48–72 h in a screw-top pressure tube, until TLC indicated complete disappearance of the starting dibromide (Figure 2). After that, the reaction mixture was cooled and the solvent was evaporated to furnish a crude solid. This was repeatedly washed with ethyl acetate to remove any of the unreacted amine, and the residue was finally subjected to repeated crystallizations from a mixture of MeOH and ethyl acetate. This afforded a hygroscopic white solid in each case. The product yields ranged from 40–50%. The purities of these lipids were ascertained from TLC; the *R_f_* of the single spot ranged from 0.2 to 0.3 (depending on the nature of the head group and spacer) in 10∶1 CHCl_3_/MeOH. All the new gemini lipids were fully characterized by ^1^H-NMR, ^13^C-NMR, mass spectrometry and CHN analysis (Table S1). Spectroscopic and analytical data for the individual lipid are given below.

### CholG-D

^1^H-NMR (CDCl_3_, 300 MHz): *δ* 0.66 (*s*, 6H), 0.87–2.36 (*m*, 82H), 3.21 (*m*, 2H), 3.52 (*s*, 12H), 3.89 (br *m*, 8H), 4.06 (br *s*, 4H), 4.23 (br *m*, 6H), 5.37 (*d, J = *4.5 Hz, 2H). ^13^C-NMR (CDCl_3_, 75 MHz): *δ* 11.59, 18.61, 19.18, 20.83, 22.38, 22.54, 23.75, 24.02, 27.60, 27.87, 27.93, 28.16, 31.63, 31.90, 35.52, 35.78, 36.53, 36.73, 38.66, 39.18, 39.36, 42.15, 49.86, 52.69, 55.78, 56.67, 61.53, 64.72, 65.21, 79.95, 121.92, 139.64. ESI-MS: Calcd. 501.8 (M^+2^/2); found 501.2 (M^+2^/2). Anal. (C_66_H_118_Br_2_N_2_O_4_): Calcd. C, 68.13; H, 10.22; N, 2.41; found C, 67.96; H, 10.13; N, 2.47.

### CholHG-D

^1^H-NMR (CDCl_3_, 300 MHz): *δ* 0.68 (*s*, 6H), 0.87-2.36 (*m*, 82H), 3.21 (*m*, 2H), 3.54 (*s*, 6H), 3.84 (br *m*, 12H), 4.08 (br *s*, 4H), 4.21 (br *m*, 6H), 5.37 (*d, J = *4.5 Hz, 2H). ^13^C-NMR (CDCl_3_, 75 MHz): *δ* 11.66, 18.59, 19.15, 20.70, 22.29, 22.57, 23.74, 24.06, 27.73, 27.84, 27.97, 28.17, 31.66, 31.76, 35.50, 35.90, 36.47, 36.92, 38.68, 39.15, 39.37, 42.14, 47.76, 52.53, 55.73, 56.70, 61.53, 64.75, 65.24, 65.58, 79.65, 122.04, 139.69. ESI-MS: Calcd. 531.9 (M^+2^/2); found 531.6 (M^+2^/2). Anal. (C_68_H_122_Br_2_N_2_O_6_^.^H_2_O): Calcd. C, 65.78; H, 10.07; N, 2.26; found C, 65.59; H, 9.89; N, 2.31.

### CholHG-1ox

^1^H-NMR (CDCl_3_, 300 MHz): *δ* 0.68 (*s*, 6H), 0.87–2.36 (*m*, 82H), 3.21 (*m*, 2H), 3.53 (*s*, 6H), 3.87 (br *m*, 12H), 4.03 (br *s*, 4H), 4.17 (br *s*, 4H), 4.27 (br *s*, 4H), 5.37 (*d, J = *4.5 Hz, 2H). ^13^C-NMR (CDCl_3_, 75 MHz): *δ* 11.76, 18.64, 19.24, 20.76, 22.31, 22.61, 23.69, 24.11, 27.68, 27.76, 27.89, 28.09, 31.58, 31.81, 35.57, 35.87, 36.51, 36.81, 38.59, 39.25, 39.48, 42.04, 47.69, 52.63, 55.81, 56.62, 61.49, 64.68, 65.34, 65.78, 79.87, 122.17, 139.86. ESI-MS: Calcd. 523.9 (M^+2^/2); found 523.4 (M^+2^/2). Anal. (C_68_H_122_Br_2_N_2_O_5_^.^2H_2_O): Calcd. C, 65.68; H, 10.21; N, 2.25; found C, 65.42; H, 10.05; N, 2.29.

### CholHG-2ox

^1^H-NMR (CDCl_3_, 300 MHz): *δ* 0.68 (*s*, 6H), 0.87–2.36 (*m*, 82H), 3.21 (*m*, 2H), 3.51 (*s*, 6H), 3.74 (br *s*, 4H), 3.88 (br *m*, 12 H), 4.04 (br *s*, 4H), 4.13 (br *s*, 4H), 4.23 (br *s*, 4H), 5.37 (*d, J = *4.5 Hz, 2H). ^13^C-NMR (CDCl_3_, 75 MHz): *δ* 11.62, 18.77, 19.17, 20.88, 22.53, 22.79, 23.91, 24.31, 27.81, 28.19, 28.24, 30.76, 31.89, 31.72, 35.60, 36.01, 36.74, 36.91, 38.78, 39.58, 39.51, 42.27, 47.66, 52.68, 55.91, 56.65, 61.85, 64.56, 64.69, 66.03, 70.37, 79.52, 121.96, 139.53. ESI-MS: Calcd. 545.9 (M^+2^/2); found 545.2 (M^+2^/2). Anal. (C_70_H_126_Br_2_N_2_O_6_^.^H_2_O): Calcd. C, 66.22; H, 10.16; N, 2.21; found C, 66.07; H, 9.97; N, 2.29.

### CholHG-3ox

^1^H-NMR (CDCl_3_, 300 MHz): *δ* 0.68 (*s*, 6H), 0.87–2.36 (*m*, 82H), 3.21 (*m*, 2H), 3.53 (*s*, 6H), 3.69 (br *s*, 4H), 3.76 (br *s*, 4H), 3.89 (br *m*, 12 H), 4.06 (br *s*, 4H), 4.15 (br *s*, 4H), 4.22 (br *s*, 4H), 5.37 (d, *J = *4.5 Hz, 2H). ^13^C-NMR (CDCl_3_, 75 MHz): *δ* 11.84, 18.65, 19.09, 20.76, 22.48, 22.71, 23.63, 24.05, 27.71, 27.93, 28.14, 31.58, 31.81, 35.69, 36.12, 36.48, 36.85, 38.50, 39.44, 39.65, 42.19, 47.79, 52.57, 55.86, 56.63, 61.94, 64.59, 64.90, 65.87, 70.01, 70.28, 79.55, 122.23, 139.95. ESI-MS: Calcd. 567.9 (M^+2^/2); found 567.1 (M^+2^/2). Anal. (C_72_H_130_Br_2_N_2_O_7_^.^2H_2_O): Calcd. C, 64.07; H, 10.16; N, 2.08; found C, 63.86; H, 10.19; N, 2.17.

### CholHG-4ox

^1^H-NMR (CDCl_3_, 300 MHz): *δ* 0.68 (*s*, 6H), 0.87–2.36 (*m*, 82H), 3.21 (*m*, 2H), 3.51 (*s*, 6H), 3.64 (br *s*, 8H), 3.73 (br *s*, 4H), 3.85 (br *m*, 12 H), 4.07 (br *s*, 4H), 4.16 (br *s*, 4H), 4.24 (br *s*, 4H), 5.37 (d, *J = *4.5 Hz, 2H). ^13^C-NMR (CDCl_3_, 75 MHz): *δ* 11.76, 18.54, 19.29, 21.13, 22.71, 22.80, 23.77, 24.34, 27.81, 28.04, 28.22, 31.90, 31.76, 35.62, 36.07, 36.68, 36.84, 38.83, 39.37, 39.61, 42.36, 47.72, 52.74, 56.08, 56.58, 61.71, 64.67, 65.12, 65.93, 70.07, 70.31, 70.40, 79.75, 122.13, 139.68. ESI-MS: Calcd. 589.9 (M^+2^/2); found 589.5 (M^+2^/2). Anal. (C_74_H_134_Br_2_N_2_O_8_^.^H_2_O): Calcd. C, 65.46; H, 10.1; N, 2.06; found C, 65.29; H, 10.15; N, 2.16.

### Liposome Preparation

A given weight of each cationic lipid was dissolved in chloroform in separate Wheaton glass vials. Thin films were made from each vial by evaporating the organic solvent under a steady stream of dry nitrogen. Lipid membranes were hydrated, freez-thawed followed by bath sonication at 70°C for 15 min afforded cationic lipid aggregates. Sterile condition was maintained strictly while preparing the aqueous suspensions of each lipid.

### Dynamic Light Scattering (DLS)

Lipid (∼0.33 mM) and lipoplex (∼0.2 mM) suspensions were prepared in water and were used for dynamic light scattering measurements at room temperature using a Malvern Zetasizer Nano ZS particle sizer (Malvern Instruments Inc., MA), which employed an incident laser beam of ∼633 nm wavelength.

### Transmission Electron Microscopy (TEM)

Freshly prepared aqueous suspensions of the cholesteryl lipid (∼1 mM) and lipoplex (∼0.2 mM) suspensions were examined under TEM by negative staining using 0.1% uranyl acetate. The samples were observed under TEM (TECNAI T20) operating at an acceleration voltage (DC voltage) of 100 keV. Micrographs were recorded at a magnification of 4000–20000 X.

### Cast-Film XRD Measurements

The experiment was done following the reported procedures [51]. X-ray diffraction of an individual cast film was performed using the reflection method with a Phillips X-ray diffractometer. The X-ray beam was generated with a Cu anode and the Cu Kα_1_ beam of wavelength 1.5418 Å was used for the experiments. Scans were performed for 2θ values up to 14°.

### Zeta Potential Titrations

To find out the effect of DOPE inclusion on the zeta potentials of gemini lipid suspensions, various gemini lipid and gemini lipid:DOPE liposomes (0.5 mg/mL) were gradually added to aqueous solution of pEGFP-C3 plasmid DNA (4 µg/mL). Zeta potential values were recorded upon each addition. Effect of serum was observed by recording the potential by adding gemini lipid:DOPE aqueous solution of pEGFP-C3 plasmid DNA (4 µg/mL) possessing 10% FBS. All the zeta potentials were recorded at the N/P charge ratio of 0, 0.125, 0.25, 0.5, 0.75, 1 and 2.

### Ethidium Bromide Displacement Assay

Fluorescence emission due to ethidium bromide (EB) at 592 nm was monitored in a Hitachi model F-4500 spectrofluorimeter (λ_ex_ = 526 nm). Typically, emission was measured for EB (7 µM) in 5 mM HEPES, 0.1 M NaCl, pH 7.4 buffer. To this, plasmid DNA pEGFP-C3 (50 µM) was added and again emission due to EB upon complexation with duplex DNA was measured at 37°C and this was continued till saturating concentration of plasmid DNA was added to obtain maximum fluorescence emission. Then, an aliquot of a given lipid suspension (0.8 µM) was added into pre-formed EB/plasmid DNA complex in 5 mM HEPES (pH 7.4) buffer. This resulted in a fluorescence quenching. Progressive addition of SDS (4.5 µM aliquot) to cationic lipid/DNA complex (formed as described above) resulted in a release of DNA from the above complex. Due to release of DNA, EB was re-intercalated and fluorescence emission increased. If F_0_ is the fluorescence intensity (FI) of un-intercalated and F_max_ is the FI of the fully intercalated EB, and F_x_ is the FI for a given concentration of liposome or SDS, then % FI = (F_x_−F_0_)/(F_max_−F_0_) [52]. Assay was also performed in presence of 10% FBS adding 1/10 volume of FBS to the mixture of EB, DNA and liposomes.

### Plasmid DNA with Reporter Gene

We have used plasmid pEGFP-C3 (Clontech USA) and plasmid PGL-3 for the gene transfection studies. Plasmid was amplified in *Escherichia coli* (DH10α). Amplified pEGFP-C3 was extracted and purified using Qiagen Midi Prep Plasmid Purification protocol (Qiagen, Germany) and kit. The plasmid preparations showing a value of OD_260_/OD_280_>1.8 were used.

### Cell Culture

HeLa cells (Human cervical cancer cell, transformed) were cultured in Dulbecco’s Modified Eagle’s Medium (DMEM; Sigma) supplemented with centrifuged 10% fetal bovine serum (FBS) in T25 culture flasks (Nunc, Denmark) and were incubated at 37°C in a 99% humidified atmosphere containing 5% CO_2_ inflow.

### Transfection Procedure

Transfection of pEGFP-C3 reporter gene was performed using standard protocol [19], [36]. In brief, HeLa cells were grown in T25 culture flasks. Cells were seeded with 60,000 cells per well in 24-well plates in antibiotic free 10% FBS containing DMEM medium. Cells were grown till culture got ∼70% confluence. Each experiment was performed using 0.8 µg of DNA per well unless specified otherwise. Working stocks of DNA and lipid formulations were prepared in DMEM alone. Separately diluted DNA and desired amount of lipid formulations were mixed in a total volume of 200 µl of DMEM and incubated at room temperature for about 30 min to make lipoplexes. After 30 min of complexation, 200 µl of DMEM without or with 20% FBS was added to the complexes (final DNA concentration = 12.1 µM). These lipoplex suspensions were used for transfection in absence of serum (−FBS−FBS) or presence of 10% serum (−FBS+FBS), respectively. Old medium was removed from the wells and washed properly with DMEM alone. Lipid-DNA complexes were added to the cells. Cells were incubated for 6 h in presence of lipoplexes. Similarly, for transfections in presence of 20%-50% of serum, pre-formed lipoplexes were diluted with 200 µl DMEM containing 40%–100% FBS.

After 6 h of incubation, old medium was removed and cells were washed with DMEM and 500 µl of 10% FBS containing DMEM was added. Cells were harvested for reporter gene assay 48 h post transfection. For pEGFP-C3 reporter gene expression assay, cells were washed with DPBS buffer and trypsinized with trypsin. Trypsinized cells were collected in 5% FBS containing DPBS and assayed through FACS. Positive control transfections were performed in each case using commercial transfection reagent ‘Lipofectamine 2000’ using standard conditions specified by the manufacturer protocol (not shown). All the experiments were performed in duplicates and results presented are the average of at least two such independent experiments performed on two different days.

### Flow Cytometry

The reporter gene expression was examined by fluorescence microscopy at regular intervals and was quantified 42 h post-transfection by flow cytometry. Cells were trypsinized with 100 µl of 1% trypsin (EDTA 0.02%, dextrose 0.05%, and trypsin 0.1%) and collected in centrifuge tubes in 200 µl of 5% FBS containing DPBS. Duplicate cultures were pooled and quantified immediately using Becton and Dickinson flow cytometer equipped with a fixed laser source at 488 nm [53]. Here, % GFP cells represent the percentage of cells expressed green fluorescence protein whereas MFI represents the mean of the amount of GFP expressed in all the cells.

### Luminometry

Finally the best transfection agent emerged out of the present study was compared with a few other commercially available transfecting agents. For PGL-3 reporter gene expression assay, after performing the same process as performed for pEGFP-C3, cells were washed with DPBS and lysed with 1x lysis buffer (Promega). Gene expression was assayed through luciferase assay using LAR II reagent (Promega) in equal volume with protein solution and monitored based on the reading on an illuminometer.

### Cytotoxicity Assay

Cytotoxicity of each lipid formulation toward HeLa cells was determined using 3-(4,5-dimethylthiazole-2-yl)-2,5-diphenyltetrazolium bromide reduction assay (MTT assay) following literature procedure [54]. Nearly 15,000 cells were plated in 96-well plates (Nunc, Denmark) and grown till ∼70% confluence. Lipid-DNA complexes were prepared using 0.2 µg of DNA per well with variation in N/P charge ratio. Lipoplexes were incubated with cells for 6 h. Cell were washed and re-incubated with 10% FBS containing growth medium. After 42 h, 20 µl of MTT was added to each well and the mixtures were further incubated for 4–5 h. Blue formazan crystals were seen when checked under microscope. Media were removed and 200 µl of DMSO was added per well and kept on flat rocker for 10 min to dissolve the formazan crystals. The absorbance was measured using a microtiter plate reader. The % cell viability was then calculated from readings obtained from ELISA reader using formula, % Viability = [{A590- (treated cells) – (background)}/{A590 (untreated cells) – background}] x 100.

### Bromodeoxyuridine (BrDU) Incorporation Assay

A 5-bromo-28-deoxyuridine cell proliferation kit (cat. no. QIA58) was obtained from Calbiochem®. To determine the index of DNA synthesis and cell proliferation, BrdU was measured according to the instructions of the manufacturer. Briefly, 6 x10^3^ cells/well were plated in each well of a 96-well tissue culture plate. Medium supplemented with 10% FBS was added and cells were allowed to adhere for 24h. Cells were serum deprived for 12h to slow down the proliferation. Further, cells were incubated with optimized concentrations and N/P charge ratios of liposomes and lipoplexes for 12h in ambient conditions. At the end of the incubation period, cells were co-incubated with 1∶2000 dilution of BrdU. 20 µl of the resulting mixture was added to each of liposome and lipoplex formulations containing 200 µl of cell culture medium per well. Control cells were untreated and unlabeled with BrdU. Cultures were co-incubated in presence of BrdU for 4 h. After this period, cells were fixed with 200 µl fixative solution at room temperature (RT) for 30 min, washed three times with PBS at rt, and incubated with Anti-BrdU antibody (1∶500 dilution) (diluted with 1X PBS) for 2h at rt. At the end of the incubation, cells were washed three times with 300 µl 1X PBS and incubated with 100 µl (1∶1000 dilution in 1X PBS) peroxidase goat Anti-Mouse IgG HRP conjugate. After 1h of incubation, cells were washed three times with 300 µl 1X PBS. Finally, 100 µl of substrate solution was added to each well and incubated in dark for 15 min followed by quenching the reaction using 100 µl of 2.5 N H_2_SO_4_. Absorbance was measured using spectrophotometric plate reader at dual wavelengths of 450 and 595 nm. Lipoplexes were prepared using 0.8 µg DNA and each lipid with optimized DOPE to get N/P ratio 0.5, 0.5, 0.75, 0.75, 1, 1 and 4 respectively whereas liposomes were compared of only lipid and DOPE.

### Gel Electrophoresis

DNA (0.2 µg/well) was complexed with lipid formulations at different N/P charge ratios. After 30 min of complexation at room temperature, complexes were loaded on the gel and ran electrophoretically. For determining SDS induced DNA release and FBS stability, SDS or FBS was added separately and progressively to preformed lipoplex suspensions as SDS/lipid ratios and concentrations of FBS were increased progressively. For lipoplex stability determination in presence of DNase I, pre-complexed lipoplexes were incubated with enzyme DNase I for different time intervals, in absence and presence of FBS or equivalent amount of BSA. Uncomplexed DNA was run as a control for the experiment. Final observation of the gel under UV light showed bright fluorescent bands due to DNA-EB complexes. Bands outside the wells showed uncomplexed DNA while complexed DNA remained inside the well [55], [56].

### Fluorescence Microscopy of Transfection

To observe the gene expression efficiency, we used fluorescence microscopy (IX81, Olympus). This was quantified using FACS. The GFP-expressing cells were visualized under a fluorescence microscope and enumerated on FACS without fixation. Experiments were performed same as described in transfection section in 50% serum with –FBS+FBS and +FBS+FBS conditions using 0.8 µg of DNA while positive control Effectene was used as described by manufactures in presence of –FBS+FBS and +FBS+FBS (50%).

### Confocal Studies

We performed confocal microscopy on pEGFP-c3 transfected HeLa cells, exactly the same way as transfection experiments were performed. In brief, cells were plated on glass slips placed in well of 12-well plates. Cells were grown till cell-monolayer gained ∼70% confluence. The experiments were performed using 0.8 µg DNA and 1.2 µg of DNA per well. Working stocks of lipoplexes were prepared in DMEM with (−FBS+FBS) and without (–FBS−FBS) serum by conventional method. Cells were treated with these lipoplexes for 6h followed by 42h incubation in presence of 10% FBS containing DMEM. Control experiments were performed in each case by using a commercially available transfection reagent, Effectene using protocol specified by the manufacturers, in absence (−FBS−FBS) and presence (−FBS+FBS) of serum. After 42 h of incubation, samples were processed and finally observed under confocal microscope [57].

### Statistical Analysis

Statistical significance of differences between control and samples were evaluated using one-way ANOVA using GraphPad Prizm 5.0 with Dunnett or Bonferroni analysis wherever applicable. Results were considered statistically significant when the *p* value was less than 0.05.

## Supporting Information

Figure S1 **Representative negative-stain transmission electron micrographs of aqueous suspensions of lipoplexes.** **(A)** CholHG-1ox (lipid/DOPE  = 1∶4 and N/P  = 0.5∶1); **(B)** CholHG-3ox (lipid/DOPE  = 1∶2 and N/P  = 0.75∶1) and **(C)** CholHG-D (lipid/DOPE  = 1∶2 and N/P  = 1∶1).
(TIF)

Figure S2 **Hydrodynamic diameters of formulations.** Histogram showing the hydrodynamic diameters of lipid-DOPE coliposomes at optimized lipid/DOPE ratio and lipoplexes at optimized N/P ratio.
(TIF)

Figure S3 **Variation in Zeta potential on inclusion of DOPE and FBS percentage in representative lipids CholHG-1ox and Chol-3ox.** Experiment was performed using 4 µg of pEGFP-C3/mL of aqueous medium in which individually (A) CholHG-1ox, CholHG-1ox:DOPE (1∶4), CholHG-1ox:DOPE:FBS and (B) CholHG-3ox, CholHG-3ox:DOPE (1∶4), CholHG-3ox:DOPE:FBS were added gradually to vary the N/P charge ratio from 0.125 to 2.
(TIF)

Figure S4 **Lipid:DOPE molar ratio optimization for achieving highest transfection efficiency while keeping N/P ratio fixed at 0.5 in absence of serum (−FBS−FBS).** Formulations were screened for 5 different ratios from 1∶0 to 1∶4. (**A**) CholHG-1ox; (**B**) CholHG-2ox; (**C**) CholHG-3ox; (**D**) CholHG-4ox; (**E**) CholG-D and (**F**) CholHG-D. Concentration of the DNA  = 0.8 µg/well. Data are expressed as number of transfected cells and MFI as obtained from flow cytometry analysis.
(TIF)

Figure S5 **Lipid:DOPE molar ratio optimization for highest transfection efficiency possible while N/P ratio was 0.5 in presence of serum (−FBS+FBS).** Formulations were screened for 5 different ratios from 1∶0 to 1∶4. (**A**) CholHG-1ox; (**B**) CholHG-2ox; (**C**) CholHG-3ox; (**D**) CholHG-4ox; (**E**) CholG-D and (**F**) CholHG-D. Concentration of the DNA  = 0.8 µg/well. Data are expressed as number of transfected cells and MFI as obtained from flow cytometry analysis.
(TIF)

Figure S6 **Optimization of N/P charge ratio to achieve highest transfection efficiency at the optimized lipid: DOPE ratio in absence of serum (−FBS−FBS).** Formulations were screened for different N/P ratios from 0.125 to 3 to obtain maximum transfection efficiency. (A) CholHG-1ox, (B) CholHG-2ox, (C) CholHG-3ox, (D) CholHG-4ox, (E) CholG-D and (F) CholHG-D. Concentration of the DNA = 0.8 µg/well. Data are expressed as number of transfected cells and MFI as obtained from the flow cytometric analysis.
(TIF)

Figure S7 **Optimization of the N/P charge ratio to achieve highest transfection efficiency. Optimized lipid: DOPE ratios were used in serum (−FBS+FBS).** Formulations were screened for different N/P ratios from 0.125 to 3 to obtain maximum transfection efficiency. (A) CholHG-1ox, (B) CholHG-2ox, (C) CholHG-3ox, (D) CholHG-4ox, (E) CholG-D and (F) CholHG-D. Concentration of the DNA = 0.8 µg/well. Data are expressed as number of transfected cells and MFI as obtained from the flow cytometry analysis.
(TIF)

Figure S8 **Flow cytometric scans showing comparative green fluorescence intensity due to all negative control along with our lipoplexes.** (**A**) CholHG-1ox/pEGFP-C3 and (**B**) CholHG-3ox/pEGFP-C3 in 10% serum condition (−FBS+FBS).
(TIF)

Figure S9 **Effect of variation in the amount of pEGFP-C3 plasmid DNA on gene transfection efficiency. Experiment was performed on** CholHG-1ox/DOPE (1∶4 mole ratio) formulation at N/P ratio of 0.5 CholHG-1ox/DNA.
(TIF)

Figure S10 **pEGFP-C3 transfection in HEK293T cells.** (**A**) Comparative FACS histogram of GFP expression in HEK 293T cell lines after performing CholHG-1ox, CholHG-3ox and Effectene mediated transfection of pEGFP-C3 with various negative controls; (**B**) Bar diagrams show slightly better transfection efficiency of CholHG-1ox formulations compared to Effectene in terms of MFI and (**C**) Cell viability bar diagram of different formulations shows considerably high cell viability of HEK 293T cells in transfection conditions.
(TIF)

Figure S11 **DNase sensitivity of DNA bound to various lipid formulations in presence of 10% FBS.** Experiment was performed with 10 µg plasmid DNA per well. Lipid formulations were complexed with plasmid DNA at N/P ratio 2 for 30 min followed by complexation with FBS/BSA 10% (v/v)/(w/w), respectively. (**A**) DNase stability of lipid formulations in presence of 10% FBS. Stability of complexes after incubation for 2h (A1), 4h (A2), and 6h (A3) at 37°C using 0.25 unit of DNase I. (**B**) DNase stability of the lipid formulations in presence of 10% BSA. Stability of complexes after incubation for 2h (B1), 4h (B2), and 6h (B3) at 37°C using 0.25 unit of DNase I. Figure shows pure plasmid DNA lane (DNA), DNA/lipid complex (D/L  = 5), DNA/lipid complex incubated with DNaseI(DL/Dn), DNA/lipid FBS complex (DLF), DNA/lipid FBS complex incubated with DNaseI (DLF/Dn), DNA/lipid BSA complex (DLB), DNA/lipid BSA complex incubated with DNaseI (DLB/Dn).
(TIF)

Figure S12 **MTT assay of different gemini lipids and their lipoplexes at different charge ratios along with negative and positive controls, pEGFP-C 3 plasmid and Effectene, respectively.** Histograms show cytotoxicity of (A) liposomal suspensions; (B) Lipoplexes (C) DNA alone, Effetene alone and its complex with DNA. Experiments were performed in presence of 10% FBS condition using 0.1 µg of pEGFP-C3 plasmid/well in 96-well plates. Experiments were performed in 10% FBS using 0.1 µg of pEGFP-C3 plasmid/well in 96-well plates.
(JPG)

Figure S13 **BrdU assay of HeLa cells treated with individual liposome and lipoplex used for the transfection studies.** (A) In presence of 10% serum (−FBS+FBS) optimized transfection formulations of different liposomes and lipoplexes did not give any significant reduction in the cell proliferation while (B) in presence of 50% serum (+FBS+FBS), considerable reduction in cell proliferation was noticed. Experiment was performed using 0.8 µg DNA/well in lipoplexes.
(TIF)

Figure S14 **Transfection efficiency of pEGFP-C3 transfected HeLa cells.** This was visualized using fluorescence microscopy and quantified using FACS analysis. (A) Fold transfection efficiency; (B) FACS histogram obtained transfecting pEGFP-C3 in presence of 50% FBS (−FBS+FBS) and (C) 50% FBS (+FBS+FBS).
(TIF)

Figure S15 **Confocal images of pEGFP-C3 transfected HeLa cells (nuclear stained with PI).** HeLa cells transfected with (A) CholHG-1ox:DOPE (1∶1) in absence of serum (−FBS−FBS); (B) CholHG-1ox:DOPE (1∶1) in 10% serum (−FBS+FBS); (C) Effectene (1∶25) in absence of serum (−FBS−FBS); (D) Effectene (1∶25) in 10% (−FBS+FBS); (E) CholHG-1ox:DOPE (1∶1) in 10% serum (−FBS+FBS); (F) CholHG-1ox:DOPE (1∶1) in 50% serum (−FBS+FBS); (G) CholHG-3ox:DOPE (1∶1) in 10% serum (−FBS+FBS) and (H) CholHG-3ox:DOPE (1∶1) in 50% serum (−FBS+FBS).
(TIF)

Figure S16 Cholesterol based gemini lipid **CholHG-1ox** possessing -CH_2_-CH_2_-OH at the headgroups and one oxyethylene spacer is at least three times better transfecting agent *in vitro* than one of the best-known commercially available transfecting agents, Effectene (Eff.), in presence of high serum levels (50%).
(TIF)

Table S1 **Elemental analysis values of new cholesterol based gemini lipids.**
(DOC)
