# Supplementary figures and images for: Gene Transfection in High Serum Levels: Case Studies with New Cholesterol Based Cationic Gemini Lipids
Source: PLoS One. 2013 Jul 4;8(7):e68305. doi: 10.1371/journal.pone.0068305 (PMC3701654; doi:10.1371/journal.pone.0068305)

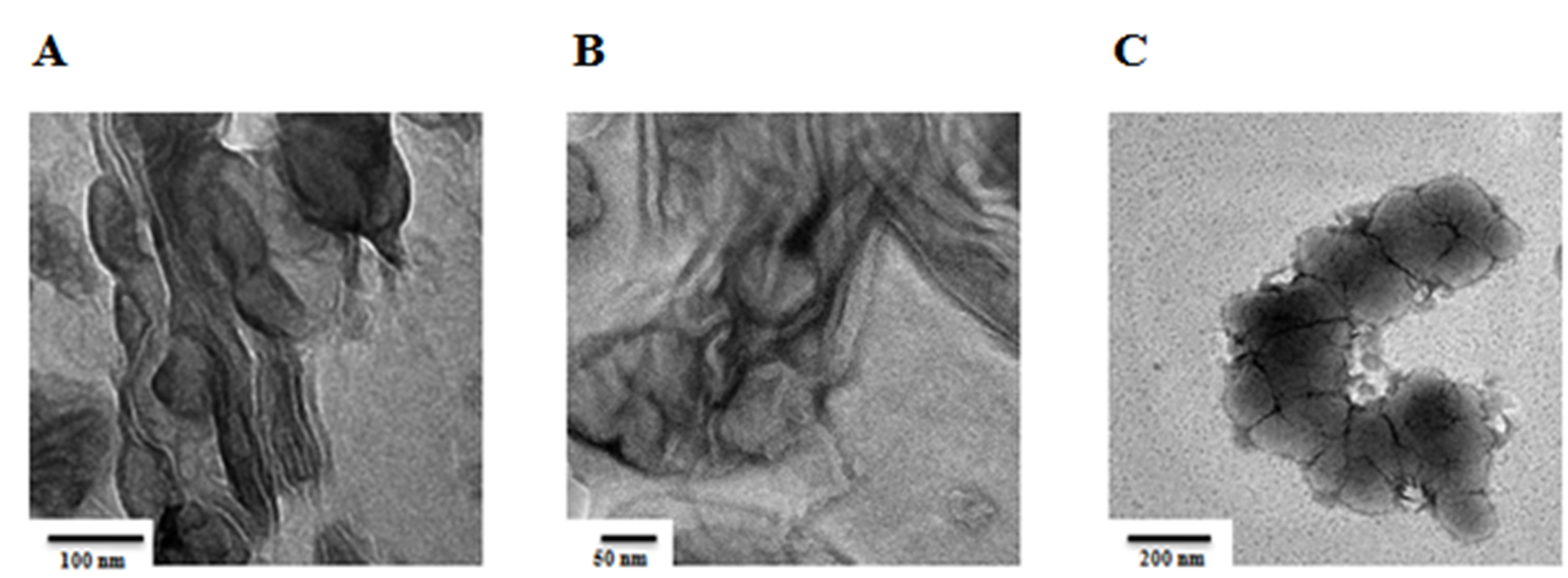

Supplement: Figure S1 — Representative negative-stain transmission electron micrographs of aqueous suspensions of lipoplexes. (A) CholHG-1ox (lipid/DOPE = 1∶4 and N/P = 0.5∶1); (B) CholHG-3ox (lipid/DOPE = 1∶2 and N/P = 0.75∶1) and (C) CholHG-D (lipid/DOPE = 1∶2 and N/P = 1∶1). (TIF) [file pone.0068305.s001.tif]

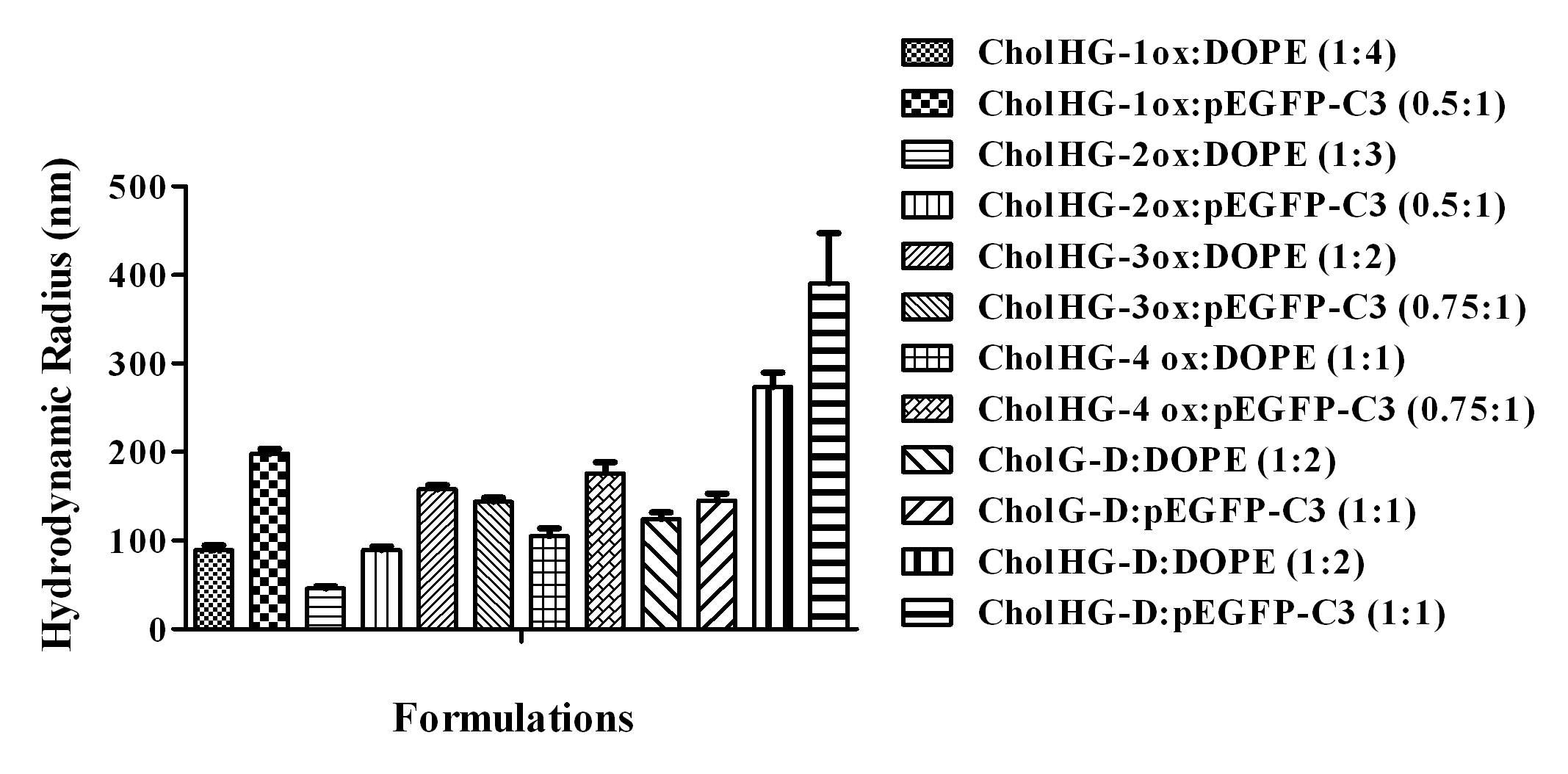

Supplement: Figure S2 — Hydrodynamic diameters of formulations. Histogram showing the hydrodynamic diameters of lipid-DOPE coliposomes at optimized lipid/DOPE ratio and lipoplexes at optimized N/P ratio. (TIF) [file pone.0068305.s002.tif]

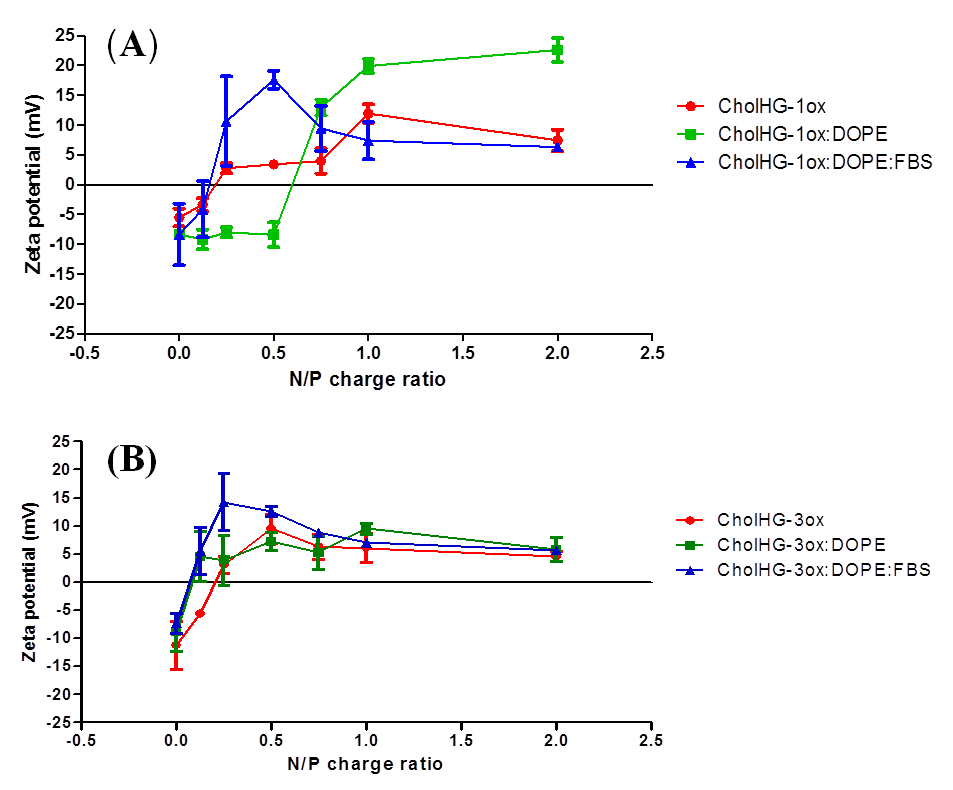

Supplement: Figure S3 — Variation in Zeta potential on inclusion of DOPE and FBS percentage in representative lipids CholHG-1ox and Chol-3ox. Experiment was performed using 4 µg of pEGFP-C3/mL of aqueous medium in which individually (A) CholHG-1ox, CholHG-1ox:DOPE (1∶4), CholHG-1ox:DOPE:FBS and (B) CholHG-3ox, CholHG-3ox:DOPE (1∶4), CholHG-3ox:DOPE:FBS were added gradually to vary the N/P charge ratio from 0.125 to 2. (TIF) [file pone.0068305.s003.tif]

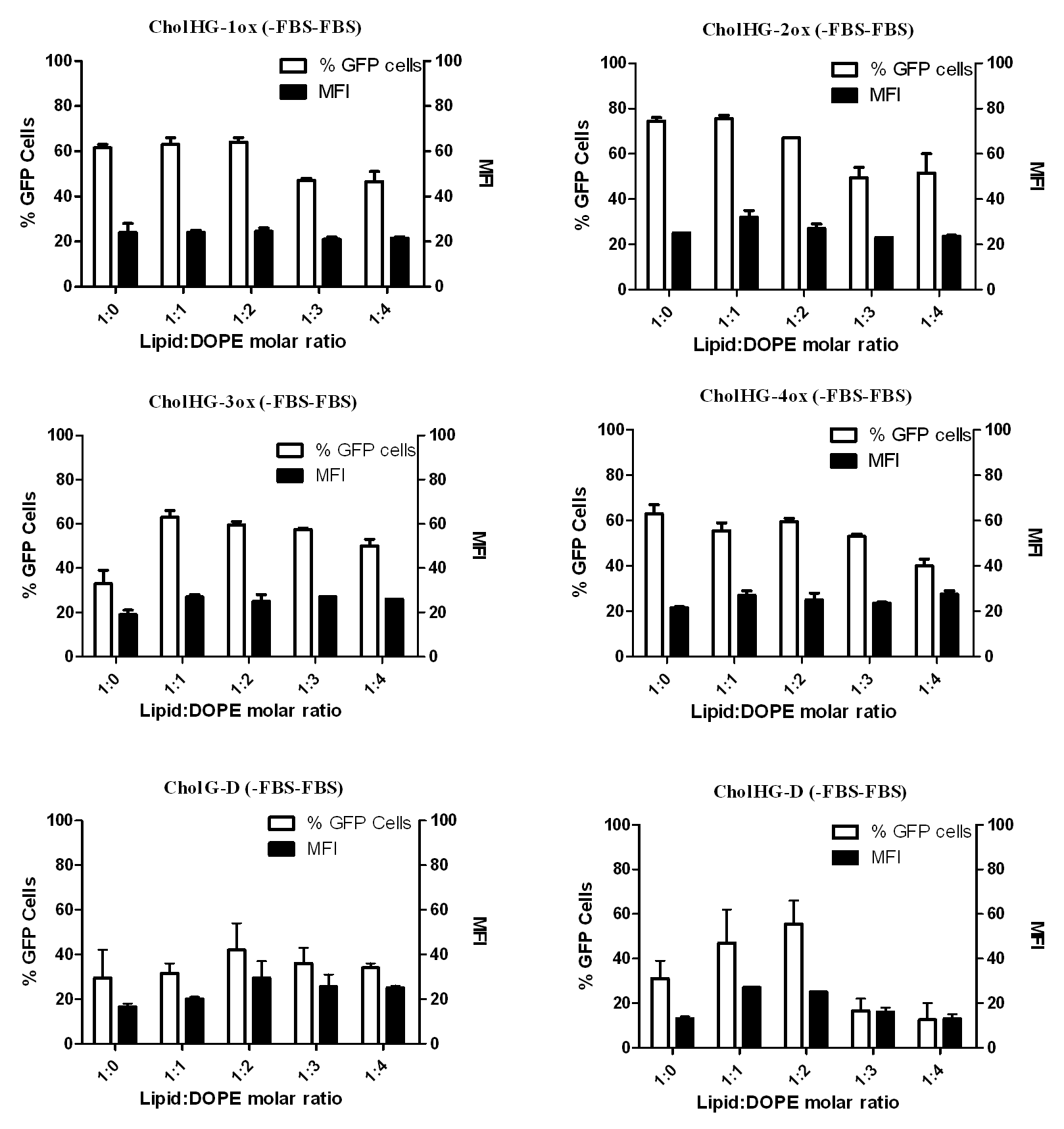

Supplement: Figure S4 — Lipid:DOPE molar ratio optimization for achieving highest transfection efficiency while keeping N/P ratio fixed at 0.5 in absence of serum (−FBS−FBS). Formulations were screened for 5 different ratios from 1∶0 to 1∶4. (A) CholHG-1ox; (B) CholHG-2ox; (C) CholHG-3ox; (D) CholHG-4ox; (E) CholG-D and (F) CholHG-D. Concentration of the DNA = 0.8 µg/well. Data are expressed as number of transfected cells and MFI as obtained from flow cytometry analysis. (TIF) [file pone.0068305.s004.tif]

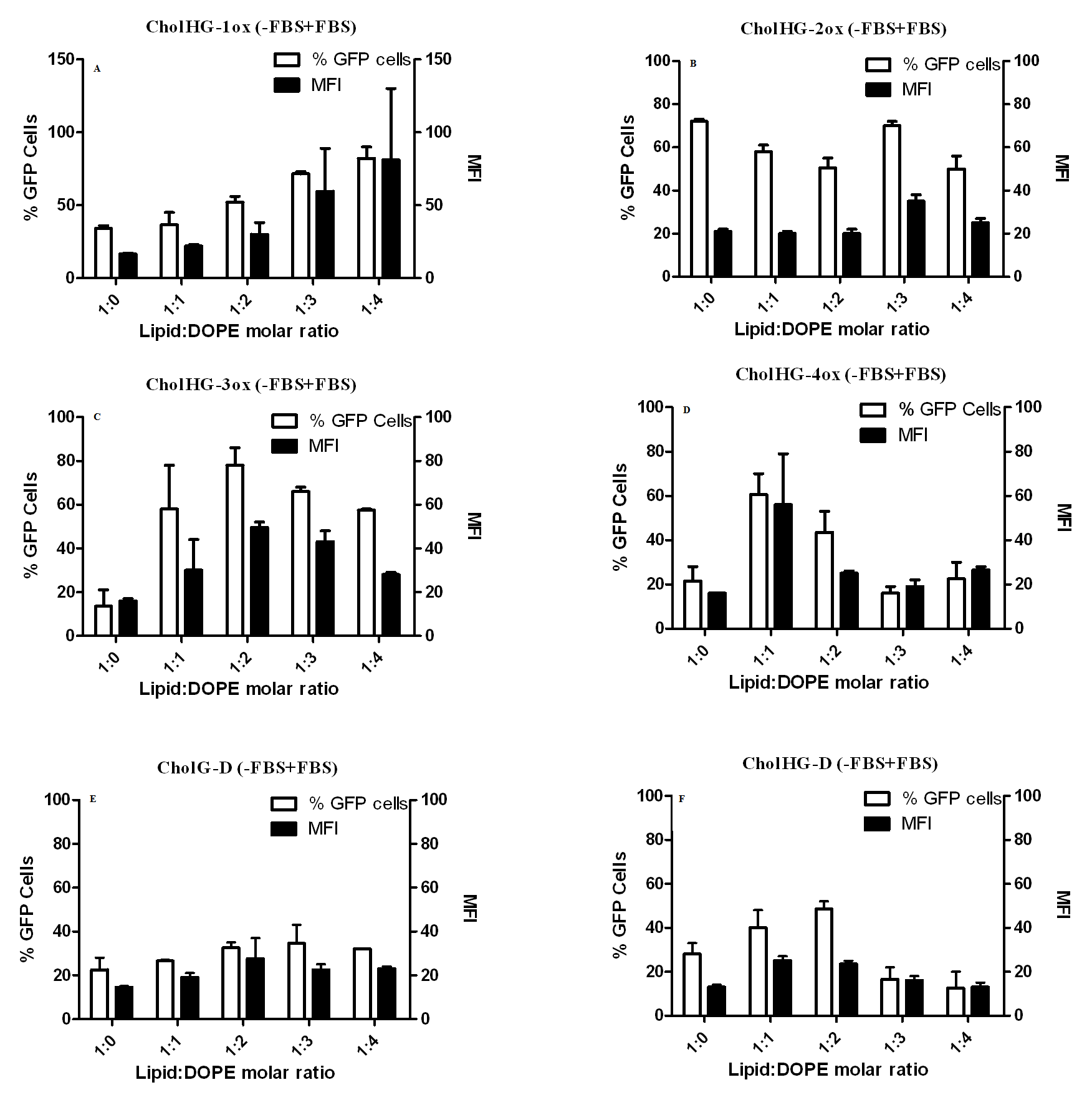

Supplement: Figure S5 — Lipid:DOPE molar ratio optimization for highest transfection efficiency possible while N/P ratio was 0.5 in presence of serum (−FBS+FBS). Formulations were screened for 5 different ratios from 1∶0 to 1∶4. (A) CholHG-1ox; (B) CholHG-2ox; (C) CholHG-3ox; (D) CholHG-4ox; (E) CholG-D and (F) CholHG-D. Concentration of the DNA = 0.8 µg/well. Data are expressed as number of transfected cells and MFI as obtained from flow cytometry analysis. (TIF) [file pone.0068305.s005.tif]

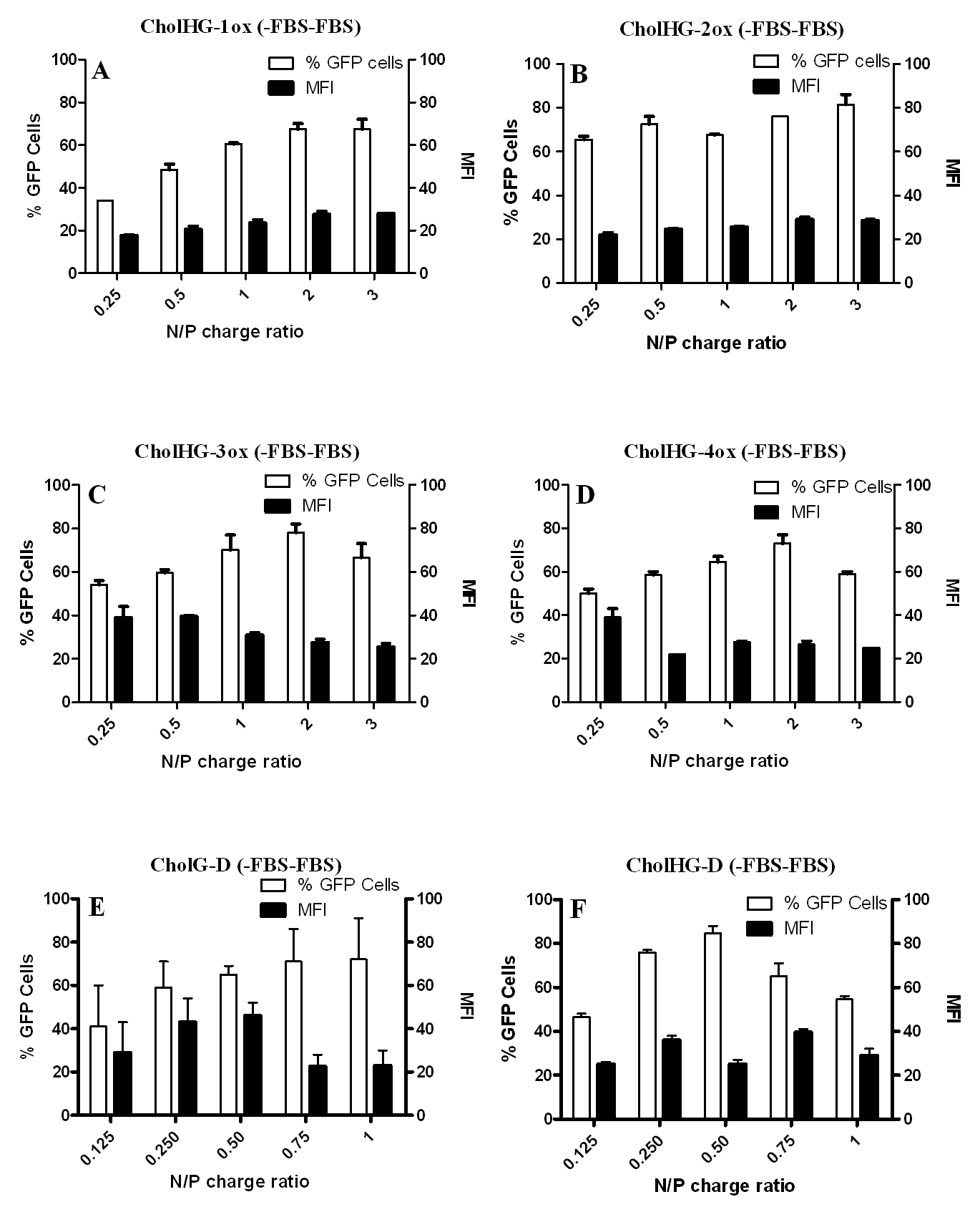

Supplement: Figure S6 — Optimization of N/P charge ratio to achieve highest transfection efficiency at the optimized lipid: DOPE ratio in absence of serum (−FBS−FBS). Formulations were screened for different N/P ratios from 0.125 to 3 to obtain maximum transfection efficiency. (A) CholHG-1ox, (B) CholHG-2ox, (C) CholHG-3ox, (D) CholHG-4ox, (E) CholG-D and (F) CholHG-D. Concentration of the DNA = 0.8 µg/well. Data are expressed as number of transfected cells and MFI as obtained from the flow cytometric analysis. (TIF) [file pone.0068305.s006.tif]

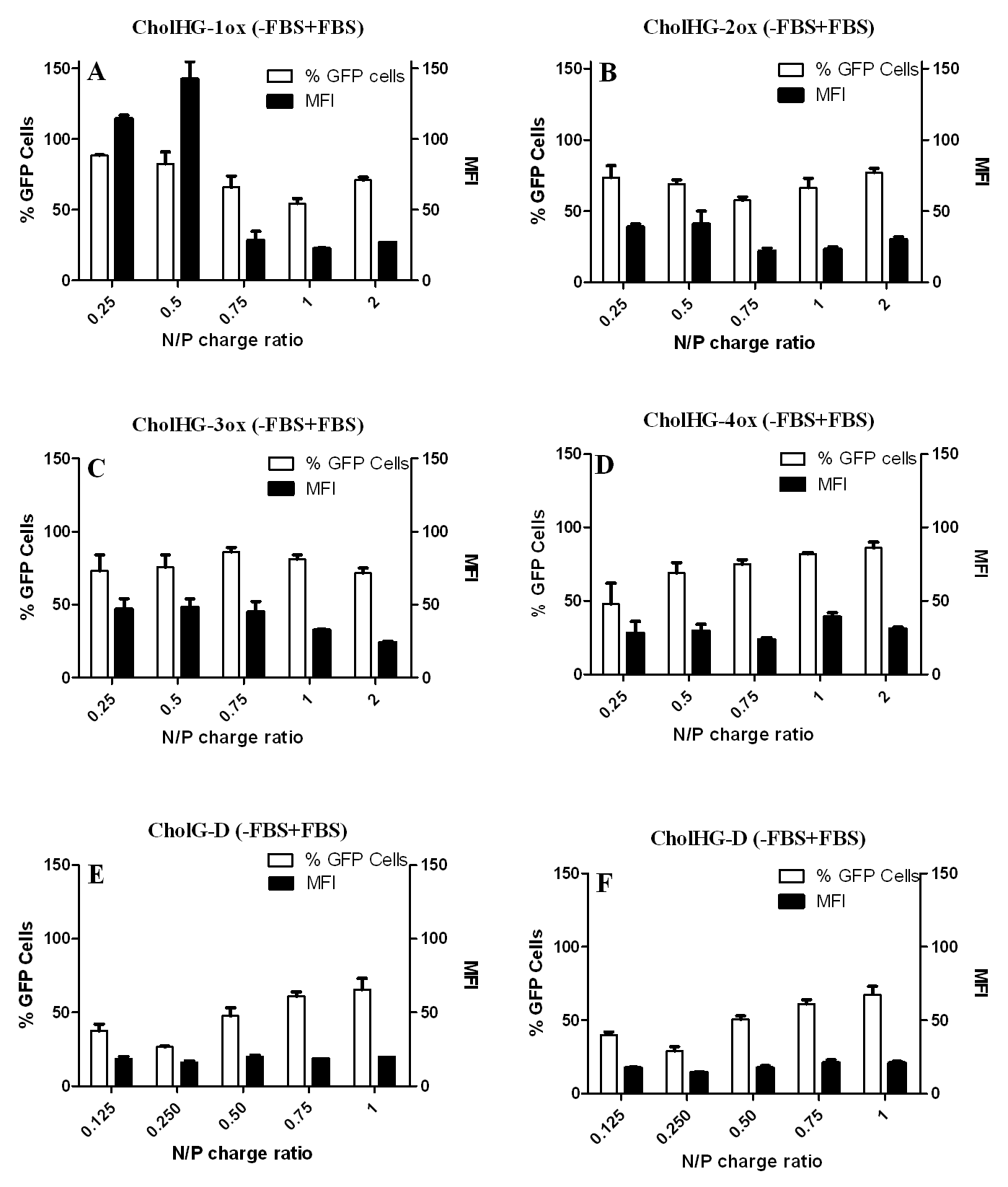

Supplement: Figure S7 — Optimization of the N/P charge ratio to achieve highest transfection efficiency. Optimized lipid: DOPE ratios were used in serum (−FBS+FBS). Formulations were screened for different N/P ratios from 0.125 to 3 to obtain maximum transfection efficiency. (A) CholHG-1ox, (B) CholHG-2ox, (C) CholHG-3ox, (D) CholHG-4ox, (E) CholG-D and (F) CholHG-D. Concentration of the DNA = 0.8 µg/well. Data are expressed as number of transfected cells and MFI as obtained from the flow cytometry analysis. (TIF) [file pone.0068305.s007.tif]

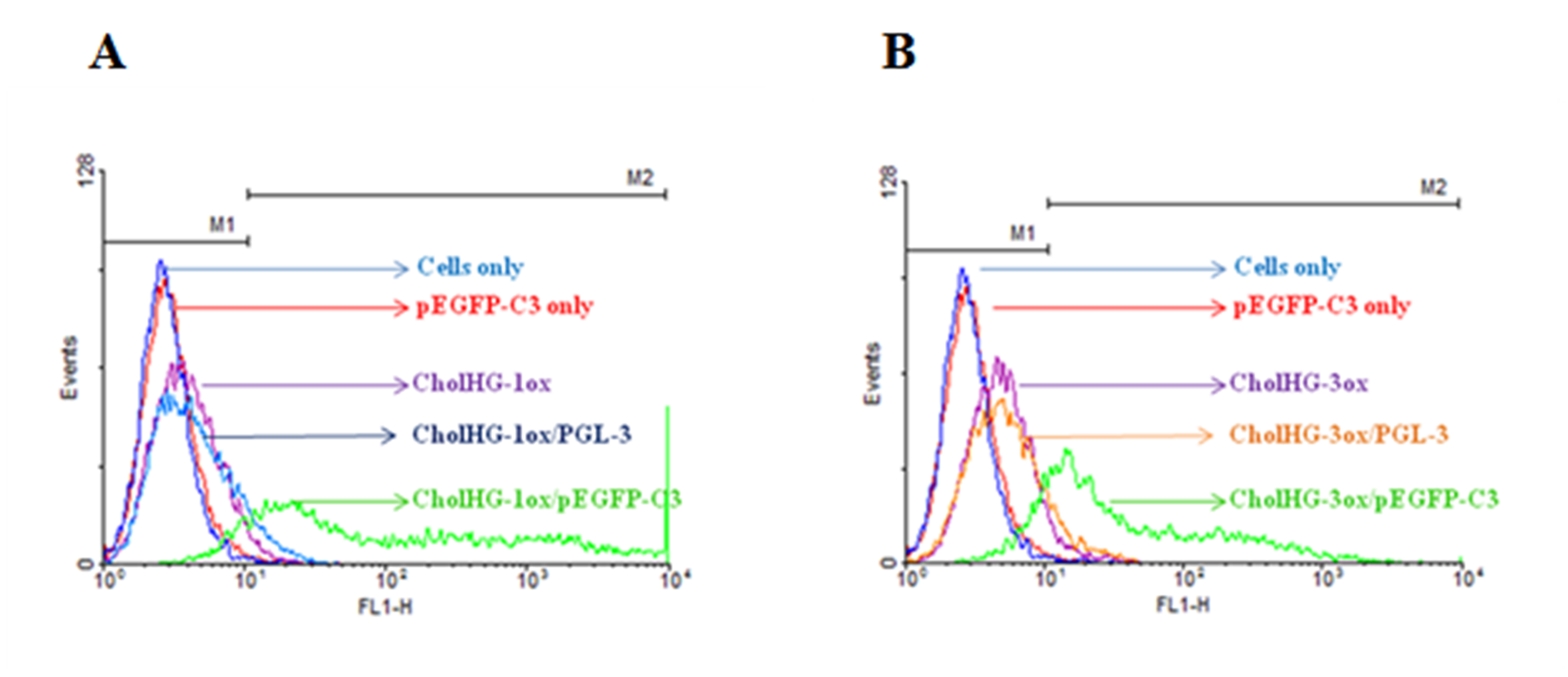

Supplement: Figure S8 — Flow cytometric scans showing comparative green fluorescence intensity due to all negative control along with our lipoplexes. (A) CholHG-1ox/pEGFP-C3 and (B) CholHG-3ox/pEGFP-C3 in 10% serum condition (−FBS+FBS). (TIF) [file pone.0068305.s008.tif]

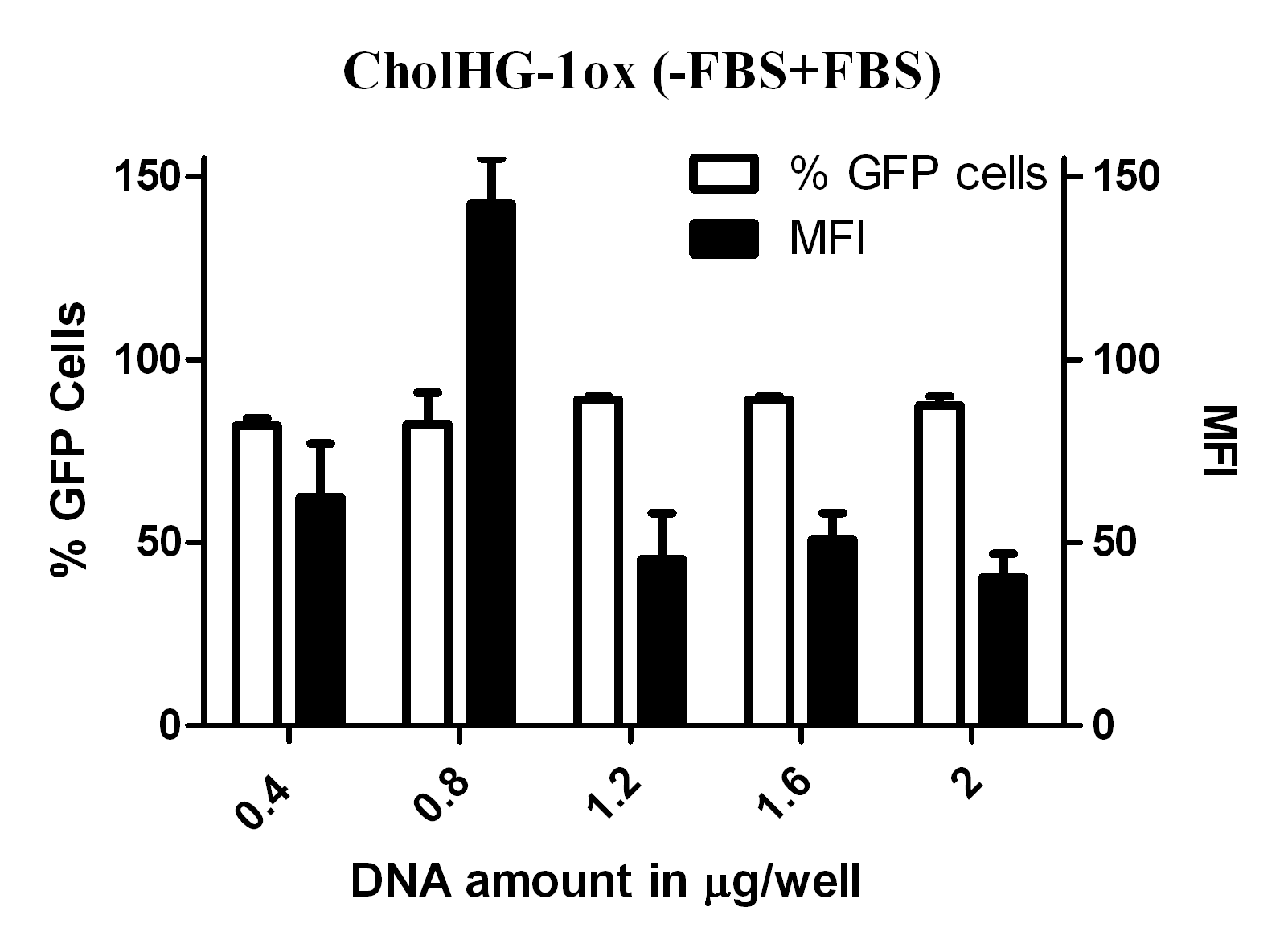

Supplement: Figure S9 — Effect of variation in the amount of pEGFP-C3 plasmid DNA on gene transfection efficiency. Experiment was performed on CholHG-1ox/DOPE (1∶4 mole ratio) formulation at N/P ratio of 0.5 CholHG-1ox/DNA. (TIF) [file pone.0068305.s009.tif]

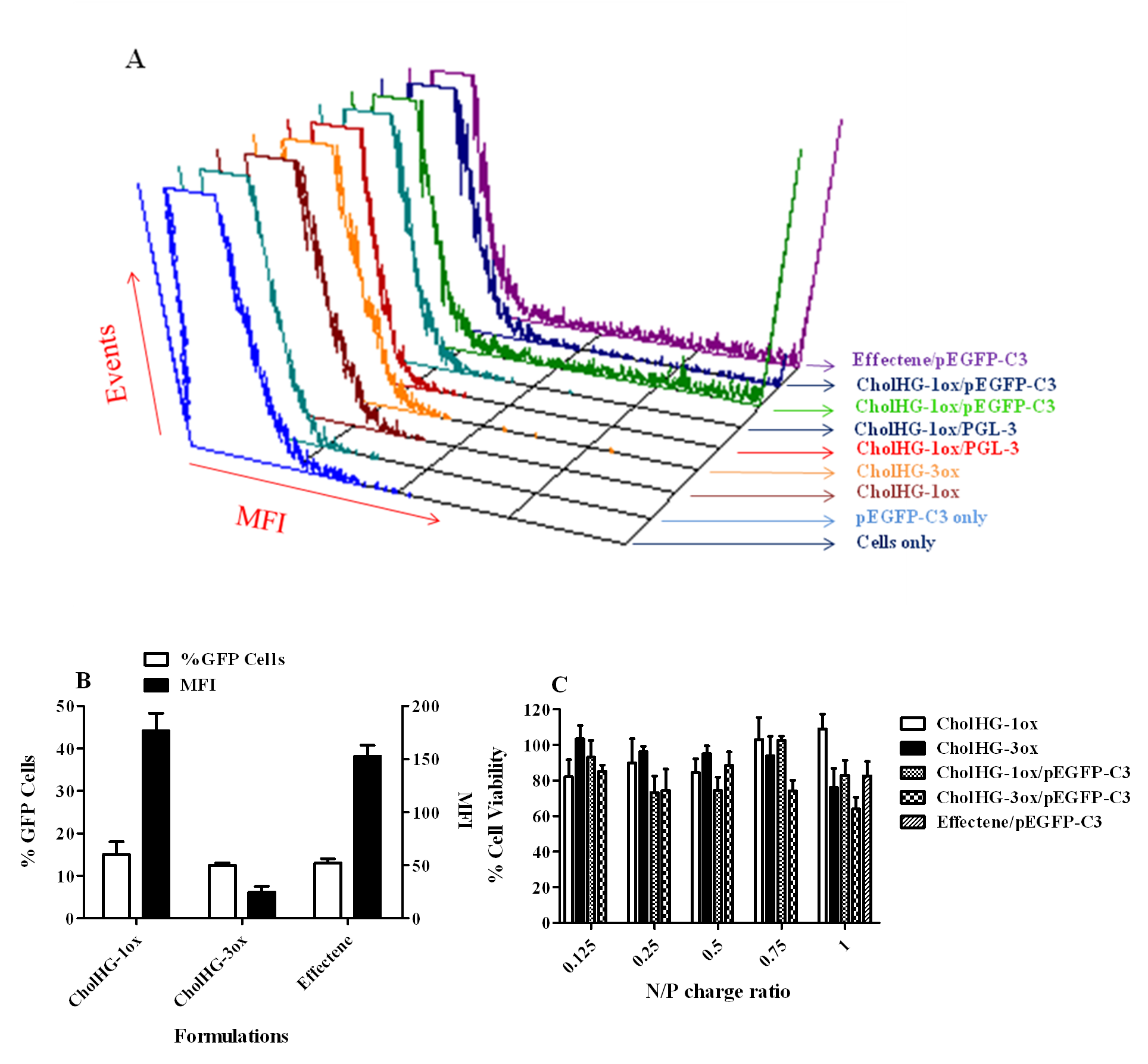

Supplement: Figure S10 — pEGFP-C3 transfection in HEK293T cells. (A) Comparative FACS histogram of GFP expression in HEK 293T cell lines after performing CholHG-1ox, CholHG-3ox and Effectene mediated transfection of pEGFP-C3 with various negative controls; (B) Bar diagrams show slightly better transfection efficiency of CholHG-1ox formulations compared to Effectene in terms of MFI and (C) Cell viability bar diagram of different formulations shows considerably high cell viability of HEK 293T cells in transfection conditions. (TIF) [file pone.0068305.s010.tif]

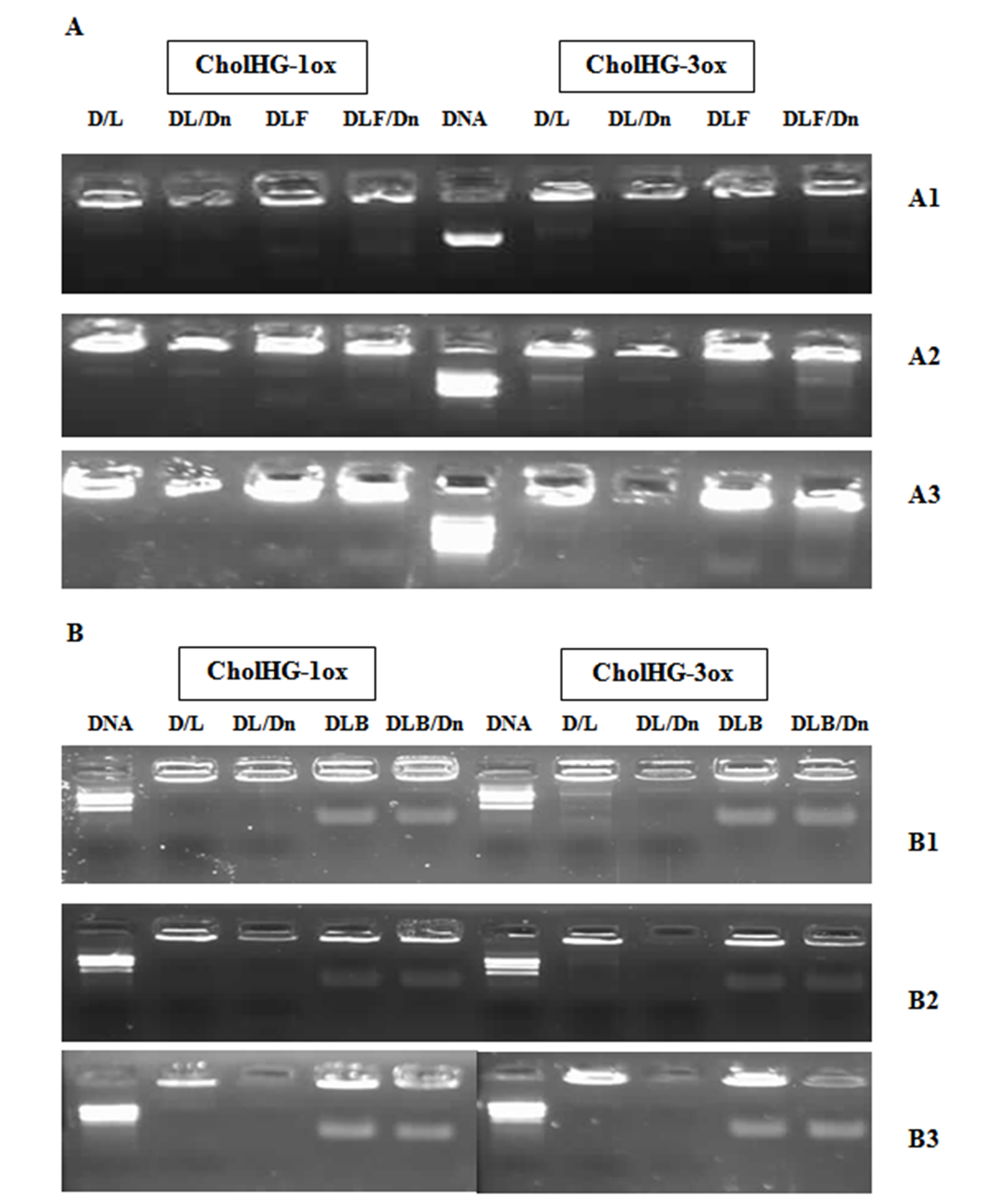

Supplement: Figure S11 — DNase sensitivity of DNA bound to various lipid formulations in presence of 10% FBS. Experiment was performed with 10 µg plasmid DNA per well. Lipid formulations were complexed with plasmid DNA at N/P ratio 2 for 30 min followed by complexation with FBS/BSA 10% (v/v)/(w/w), respectively. (A) DNase stability of lipid formulations in presence of 10% FBS. Stability of complexes after incubation for 2h (A1), 4h (A2), and 6h (A3) at 37°C using 0.25 unit of DNase I. (B) DNase stability of the lipid formulations in presence of 10% BSA. Stability of complexes after incubation for 2h (B1), 4h (B2), and 6h (B3) at 37°C using 0.25 unit of DNase I. Figure shows pure plasmid DNA lane (DNA), DNA/lipid complex (D/L = 5), DNA/lipid complex incubated with DNaseI(DL/Dn), DNA/lipid FBS complex (DLF), DNA/lipid FBS complex incubated with DNaseI (DLF/Dn), DNA/lipid BSA complex (DLB), DNA/lipid BSA complex incubated with DNaseI (DLB/Dn). (TIF) [file pone.0068305.s011.tif]

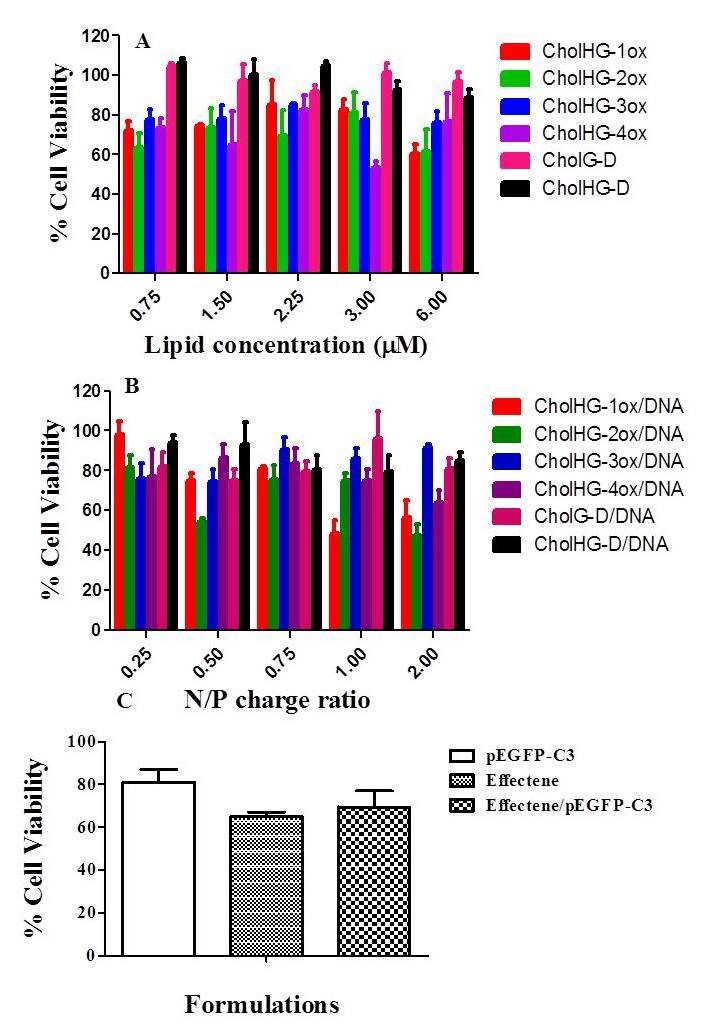

Supplement: Figure S12 — MTT assay of different gemini lipids and their lipoplexes at different charge ratios along with negative and positive controls, pEGFP-C 3 plasmid and Effectene, respectively. Histograms show cytotoxicity of (A) liposomal suspensions; (B) Lipoplexes (C) DNA alone, Effetene alone and its complex with DNA. Experiments were performed in presence of 10% FBS condition using 0.1 µg of pEGFP-C3 plasmid/well in 96-well plates. Experiments were performed in 10% FBS using 0.1 µg of pEGFP-C3 plasmid/well in 96-well plates. (JPG) [file pone.0068305.s012.jpg]

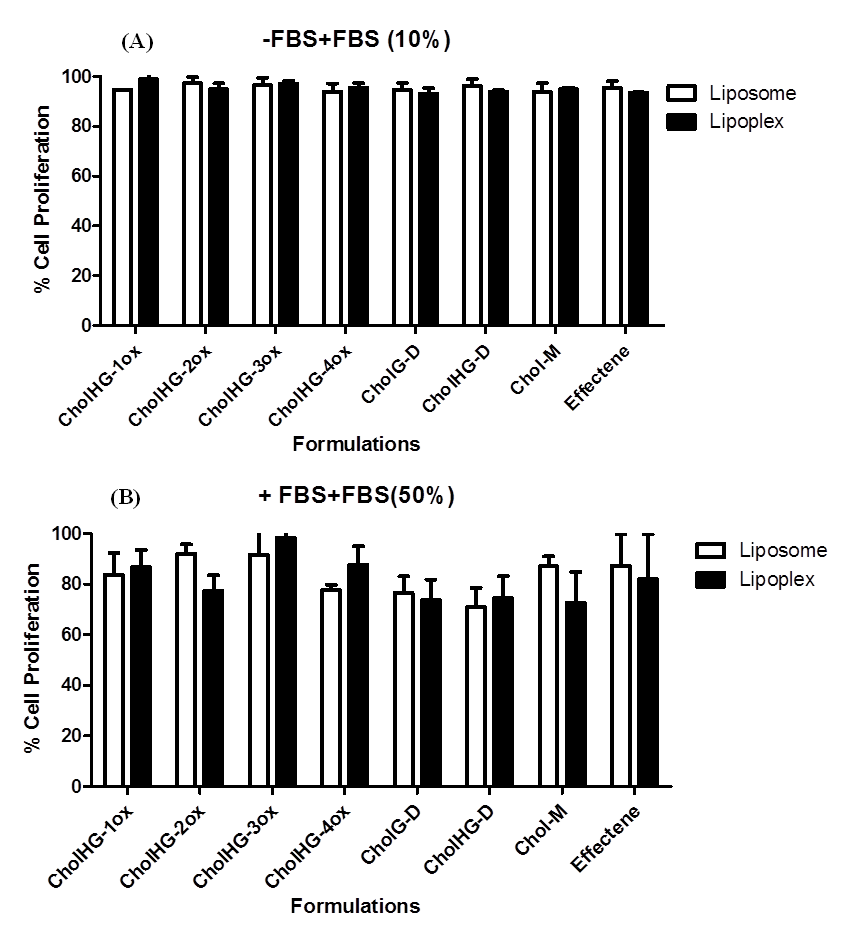

Supplement: Figure S13 — BrdU assay of HeLa cells treated with individual liposome and lipoplex used for the transfection studies. (A) In presence of 10% serum (−FBS+FBS) optimized transfection formulations of different liposomes and lipoplexes did not give any significant reduction in the cell proliferation while (B) in presence of 50% serum (+FBS+FBS), considerable reduction in cell proliferation was noticed. Experiment was performed using 0.8 µg DNA/well in lipoplexes. (TIF) [file pone.0068305.s013.tif]

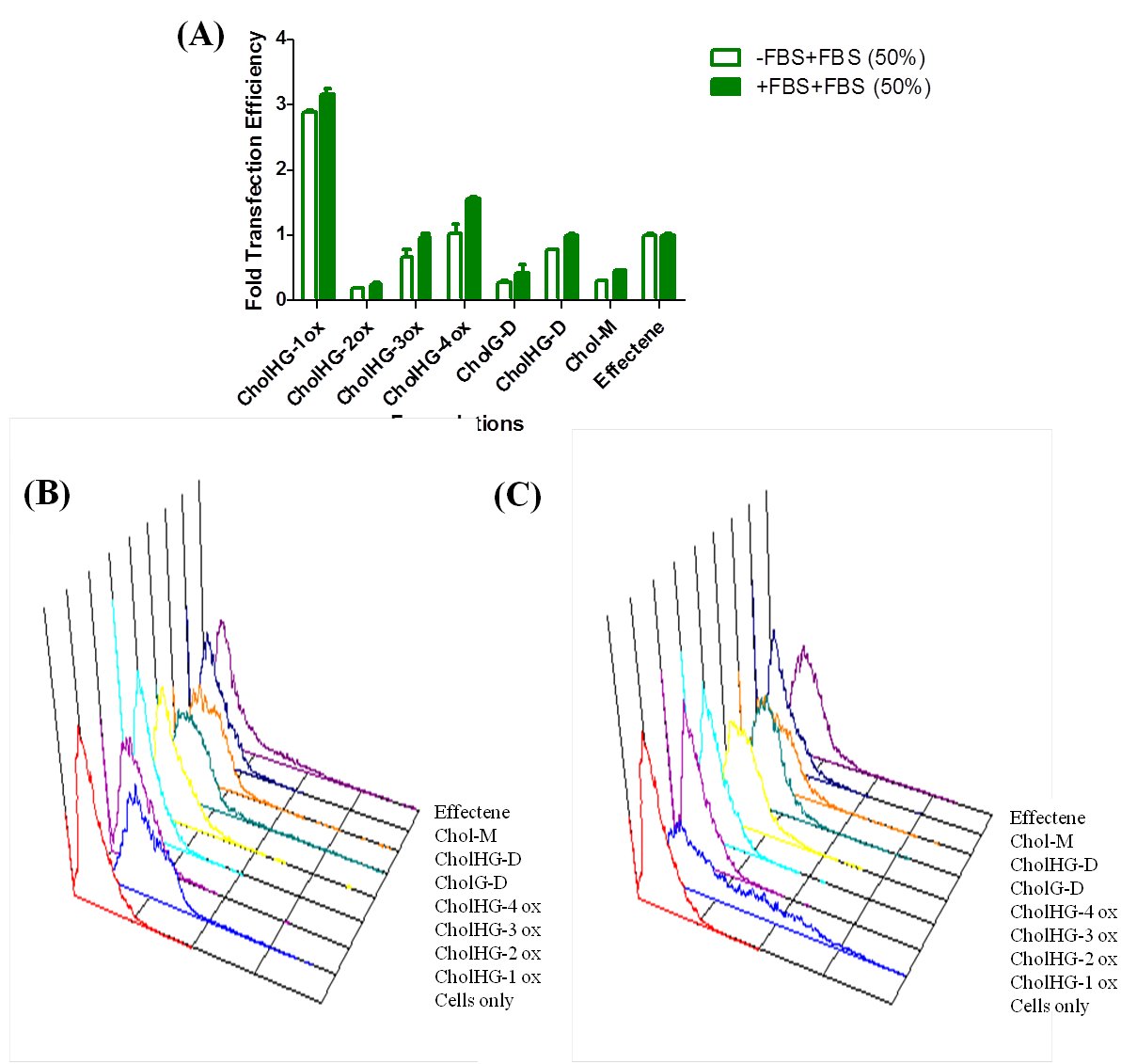

Supplement: Figure S14 — Transfection efficiency of pEGFP-C3 transfected HeLa cells. This was visualized using fluorescence microscopy and quantified using FACS analysis. (A) Fold transfection efficiency; (B) FACS histogram obtained transfecting pEGFP-C3 in presence of 50% FBS (−FBS+FBS) and (C) 50% FBS (+FBS+FBS). (TIF) [file pone.0068305.s014.tif]

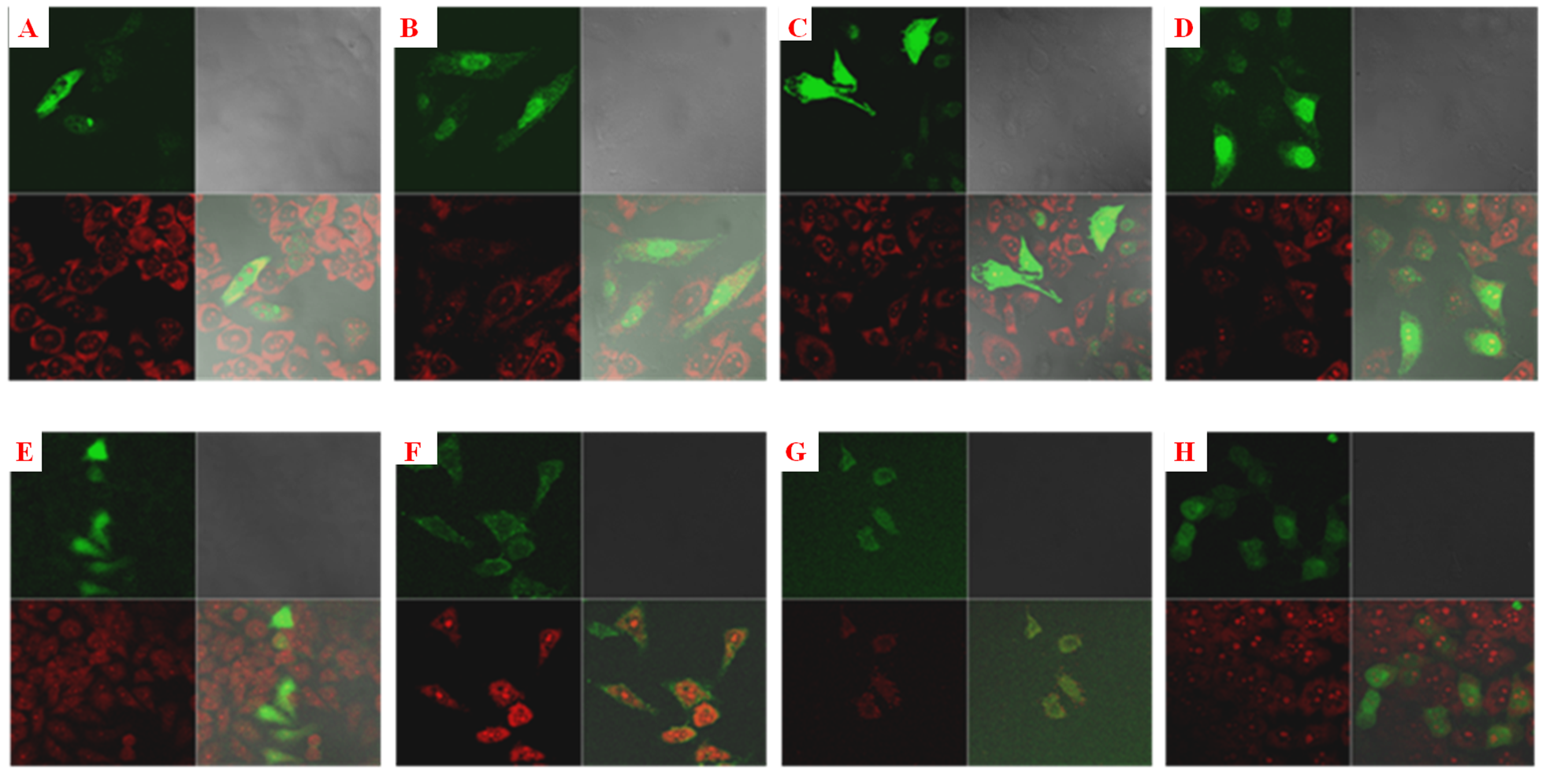

Supplement: Figure S15 — Confocal images of pEGFP-C3 transfected HeLa cells (nuclear stained with PI). HeLa cells transfected with (A) CholHG-1ox:DOPE (1∶1) in absence of serum (−FBS−FBS); (B) CholHG-1ox:DOPE (1∶1) in 10% serum (−FBS+FBS); (C) Effectene (1∶25) in absence of serum (−FBS−FBS); (D) Effectene (1∶25) in 10% (−FBS+FBS); (E) CholHG-1ox:DOPE (1∶1) in 10% serum (−FBS+FBS); (F) CholHG-1ox:DOPE (1∶1) in 50% serum (−FBS+FBS); (G) CholHG-3ox:DOPE (1∶1) in 10% serum (−FBS+FBS) and (H) CholHG-3ox:DOPE (1∶1) in 50% serum (−FBS+FBS). (TIF) [file pone.0068305.s015.tif]

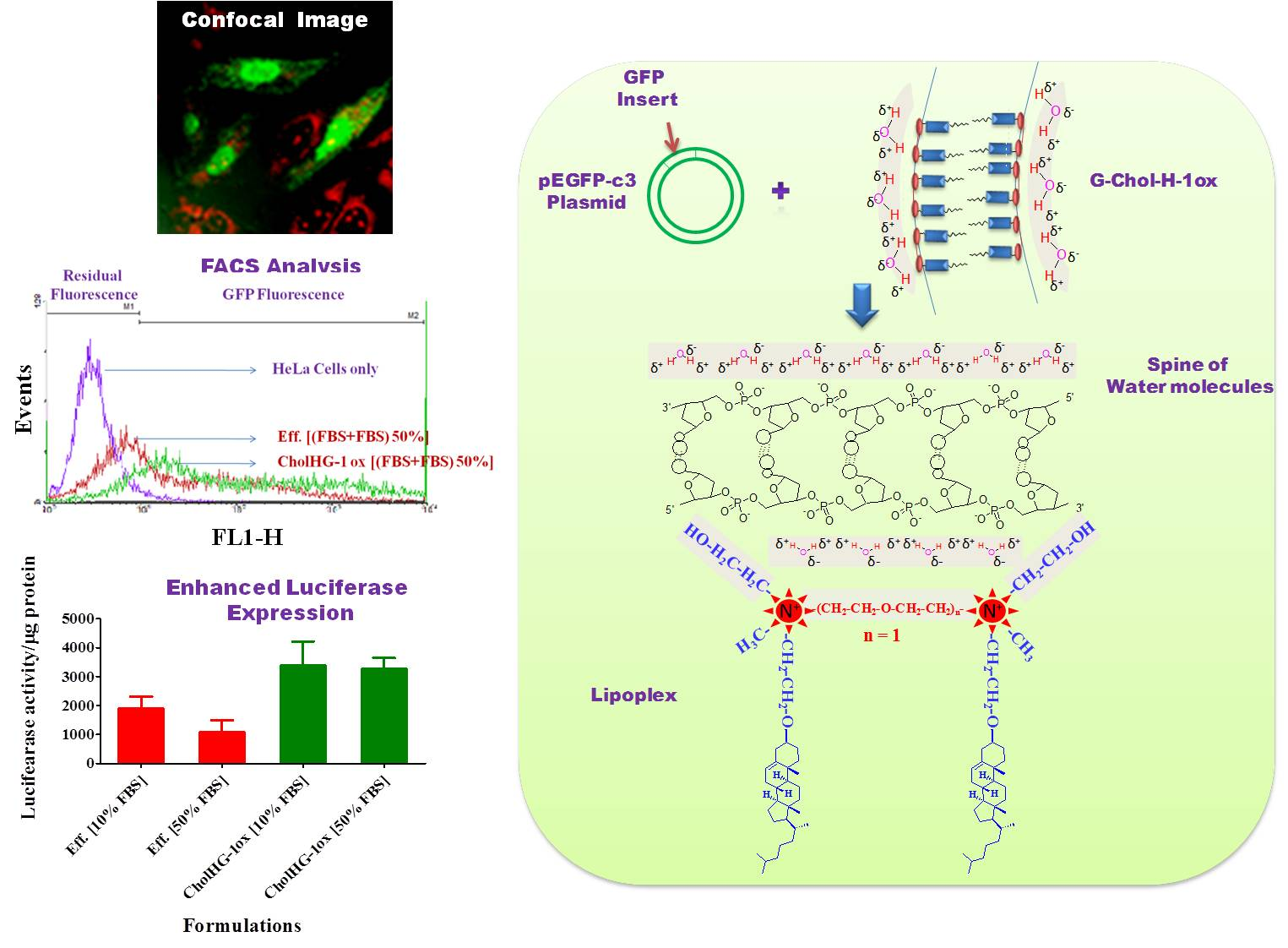

Supplement: Figure S16 — Cholesterol based gemini lipid CholHG-1ox possessing -CH2-CH2-OH at the headgroups and one oxyethylene spacer is at least three times better transfecting agent in vitro than one of the best-known commercially available transfecting agents, Effectene (Eff.), in presence of high serum levels (50%). (TIF) [file pone.0068305.s016.tif]
