# Supplementary material for: Gene Transfection in High Serum Levels: Case Studies with New Cholesterol Based Cationic Gemini Lipids
Source: PLoS One. 2013 Jul 4;8(7):e68305. doi: 10.1371/journal.pone.0068305 (PMC3701654; doi:10.1371/journal.pone.0068305)
Supplement: Table S1 — Elemental analysis values of new cholesterol based gemini lipids. (DOC) [file pone.0068305.s017.doc]

Gene Transfection in High Serum Levels. Case studies with New Cholesterol based Cationic Gemini Lipids

Santosh K. Misra,^a^ Joydeep Biswas,^a^ Paturu Kondaiah^b^ and Santanu Bhattacharya*^,a,c^

^a^Department of Organic Chemistry and ^b^Department of Molecular Reproduction, Development and Genetics, Indian Institute of Science, Bangalore 560 012, India.

^c^Chemical Biology Unit of JNCASR, Bangalore 560 064, India.

*Corresponding author and also J. C. Bose Fellow, DST, New Delhi, India.

Email: [sb@orgchem.iisc.ernet.in](mailto:sb@orgchem.iisc.ernet.in)

Phone: (91)-80-2293-2664; Fax: (91)-80-2360-0529.

Supporting Information

**Contents Page No.**

Elemental analysis results S2

TEM images of lipoplexes S3

Variation in Zeta potential S4

Hydrodynamic diameters of lipid-DOPE coliposomes and lipoplexes S5

Optimization of lipid:DOPE molar ratio in absence of serum S6

Optimization of lipid:DOPE molar ratio in presence of serum S7

Optimization of lipid:DOPE molar ratio in absence of serum S8

Optimization of lipid:DOPE molar ratio in presence of serum S9

Flow cytometric scans S10

Effect of variation in the amount of pEGFP-C3 plasmid DNA S11

pEGFP-C3 transfection in HEK 293T cells S12

DNase sensitivity of DNA bound to various lipid formulations S13

MTT assay of different formulations S14

BrDU assay S15

Quantification of GFP Expression S16

Confocal Images S17

Abstract Figure S18

**Table S1.** Elemental analysis values of new cholesterol based gemini lipids.

**Calculated**

**Found**

| **Lipid** | **Formula** | **C** | **H** | **N** | **C** | **H** | **N** |
| --- | --- | --- | --- | --- | --- | --- | --- |
| **CholG-D** | C_66_H_118_Br_2_N_2_O_4_ | 68.13 | 10.22 | 2.41 | 67.96 | 10.13 | 2.47 |
| **CholHG-D** | C_68_H_122_Br_2_N_2_O_6_^.^H_2_O | 65.78 | 10.07 | 2.26 | 65.59 | 9.89 | 2.31 |
| **CholHG-1ox** | C_68_H_122_Br_2_N_2_O_5_^.^2H_2_O | 65.68 | 10.21 | 2.25 | 65.42 | 10.05 | 2.29 |
| **CholHG-2ox** | C_70_H_126_Br_2_N_2_O_6_^.^H_2_O | 66.22 | 10.16 | 2.21 | 66.07 | 9.97 | 2.29 |
| **CholHG-3ox** | C_72_H_130_Br_2_N_2_O_7_^.^2H_2_O | 64.07 | 10.16 | 2.08 | 63.86 | 10.19 | 2.17 |
| **CholHG-4ox** | C_74_H_134_Br_2_N_2_O_8_^.^H_2_O | 65.46 | 10.1 | 2.06 | 65.29 | 10.15 | 2.16 |

**A B C**

**
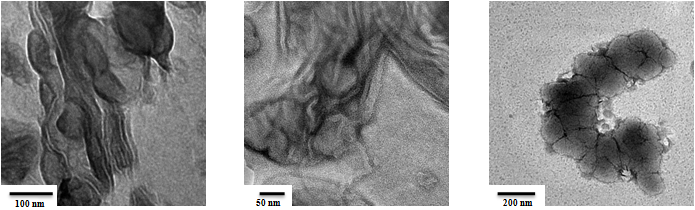
**

**Figure S1.** Representative negative-stain transmission electron micrographs of aqueous suspensions of lipoplexes of **(A)** CholHG-1ox (lipid/DOPE = 1:4 and N/P = 0.5:1); **(B)** CholHG-3ox (lipid/DOPE = 1:2 and N/P = 0.75:1) and **(C)** CholHG-D (lipid/DOPE = 1:2 and N/P = 1:1).

**Figure S2.** Histogram showing the hydrodynamic diameters of lipid-DOPE coliposomes at optimized lipid/DOPE ratio and lipoplexes at optimized N/P ratio.

**Figure S3. Variation in the Zeta potential values on inclusion of different percentage of DOPE and FBS in representative gemini lipid CholHG-1ox and Chol-3ox suspensions.** Experiment was performed using 4 µg of pEGFP-C3/mL of aqueous medium in which (A) CholHG-1ox, CholHG-1ox:DOPE (1:4), CholHG-1ox:DOPE:FBS and (B) CholHG-3ox, CholHG-3ox:DOPE (1:4), CholHG-3ox:DOPE:FBS were added gradually to vary the N/P charge ratio from 0.125 to 2.

**A**

**C**

**D**

**F**

**B**

**E**

**Figure S4.** Lipid:DOPE molar ratio optimization for achieving highest transfection efficiency while keeping N/P ratio fixed at 0.5 in absence of serum (-FBS-FBS). Formulations were screened for 5 different ratios from 1:0 to 1:4. **(A)** CholHG-1ox; **(B)** CholHG-2ox; **(C)** CholHG-3ox; **(D)** CholHG-4ox; **(E)** CholG-D and **(F)** CholHG-D. Concentration of the DNA = 0.8 μg/well. Data are expressed as number of transfected cells and MFI as obtained from flow cytometry analysis.

**e**

**Figure S5.** Lipid:DOPE molar ratio optimization for highest transfection efficiency possible while N/P ratio was 0.5 in presence of serum (-FBS+FBS). Formulations were screened for 5 different ratios from 1:0 to 1:4. **(A)** CholHG-1ox; **(B)** CholHG-2ox; **(C)** CholHG-3ox; **(D)** CholHG-4ox; **(E)** CholG-D and **(F)** CholHG-D. Concentration of the DNA = 0.8 μg/well. Data are expressed as number of transfected cells and MFI as obtained from flow cytometry analysis.

**A**

**B**

**C**

**D**

**F**

**E**

**e**

**Figure S6.** **Optimization of N/P charge ratio to achieve highest transfection efficiency at the optimized lipid: DOPE ratio in absence of serum (-FBS-FBS).** Formulations were screened for different N/P ratios from 0.125 to 3 to obtain maximum transfection efficiency. (A) CholHG-1ox, (B) CholHG-2ox, (C) CholHG-3ox, (D) CholHG-4ox, (E) CholG-D and (F) CholHG-D. Concentration of the DNA = 0.8 μg/well. Data are expressed as number of transfected cells and MFI as obtained from the flow cytometric analysis.

# Figure S7. Optimization of the N/P charge ratio to achieve highest transfection efficiency. Optimized lipid: DOPE ratios were used in serum (-FBS+FBS). Formulations were screened for different N/P ratios from 0.125 to 3 to obtain maximum transfection efficiency. (A) CholHG-1ox, (B) CholHG-2ox, (C) CholHG-3ox, (D) CholHG-4ox, (E) CholG-D and (F) CholHG-D. Concentration of the DNA = 0.8 μg/well. Data are expressed as number of transfected cells and MFI as obtained from the flow cytometry analysis.


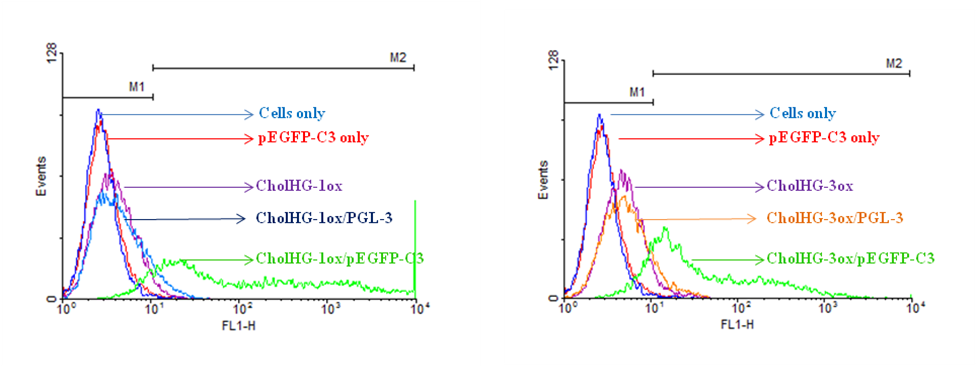
 **A B**

**Figure S8.** Flow cytometric scans showing comparative green fluorescence intensity due to all negative control along with our lipoplexes **(A)** CholHG-1ox/pEGFP-C3 and **(B)** CholHG-3ox/pEGFP-C3 in 10% serum condition (-FBS+FBS).

**Figure S9.** **Effect of variation in the amount of pEGFP-C3 plasmid DNA on gene transfection efficiency. Experiment was performed on** CholHG-1ox/DOPE (1:4 mole ratio) formulation at N/P ratio of 0.5 CholHG-1ox/DNA.

**
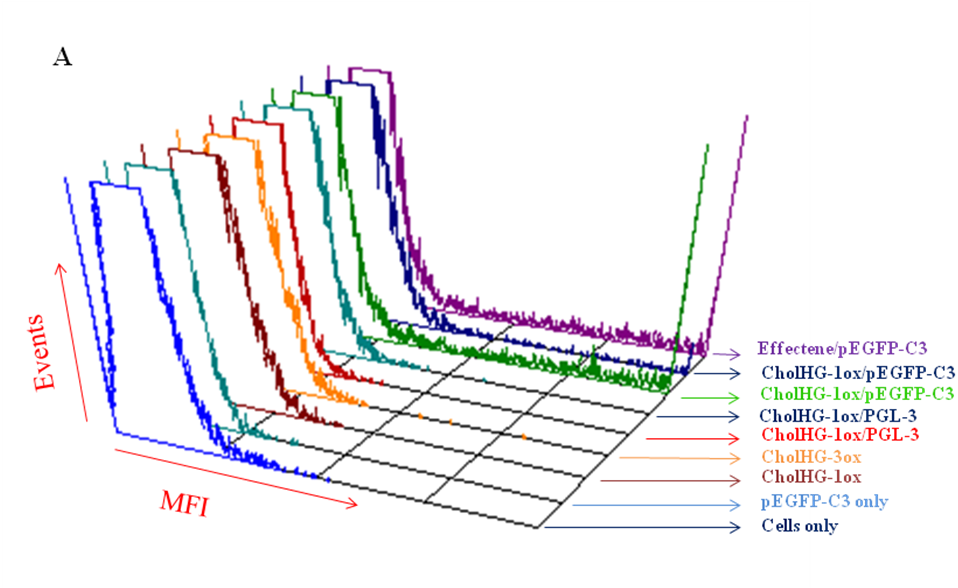
**

**Figure S10.** pEGFP-C3 transfection in HEK 293T cells. **(A)** Comparative FACS histogram of GFP expression in HEK 293T cell lines after performing CholHG-1ox, CholHG-3ox and Effectene mediated transfection of pEGFP-C3 with various negative controls; **(B)** Bar diagram shows slightly better transfection efficiency of CholHG-1ox formulations compare to Effectene in terms of MFI and **(C)** Cell viability bar diagram of different formulations shows considerably high cell viability of HEK 293T cells in transfection conditions.

**A**

**CholHG-1ox**

**CholHG-3ox**


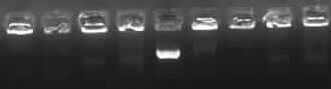

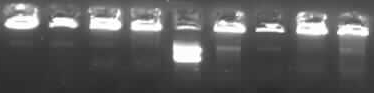

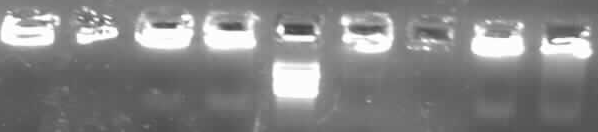


**D/L DL/Dn DLF DLF/Dn DNA D/L DL/Dn DLF DLF/Dn**

**A1**

**A2**

**A3**

**B**

**CholHG-1ox**

**CholHG-3ox**

**DNA D/L DL/Dn DLB DLB/Dn DNA D/L DL/Dn DLB DLB/Dn**


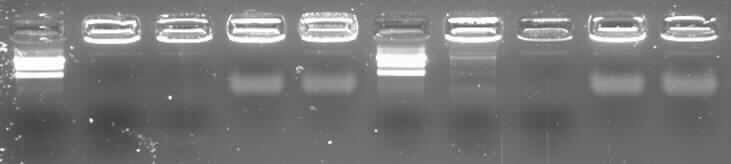

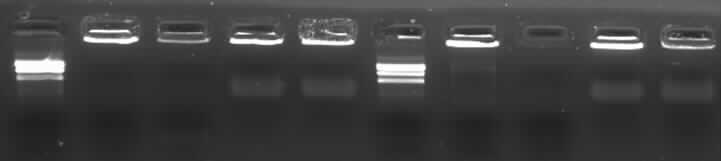

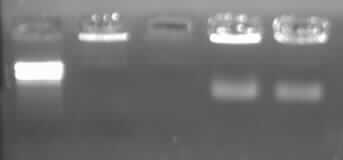

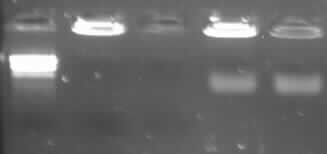


**B1**

**B2**

**B3**

**Figure S11.**  DNase sensitivity of DNA bound to various lipid formulations in presence of 10% FBS. Experiment was performed with 10µg plasmid DNA per well. Lipid formulations were complexed with plasmid DNA at N/P ratio 2 for 30 min followed by complexation with FBS/BSA 10% (v/v)/(w/w), respectively. **(A)** DNase stability of lipid formulations in presence of 10% FBS. Stability of complexes after incubation for 2h (A1), 4h (A2), and 6h (A3) at 37 ^o^C using 0.25 unit of DNase I. **(B)** DNase stability of lipid formulations in presence of 10% BSA. Stability of complexes after incubation for 2h (B1), 4h (B2), and 6h (B3) at 37 ^o^C using 0.25 unit of DNase I. Figure shows pure plasmid DNA lane (DNA), DNA/lipid complex (D/L = 5), DNA/lipid complex incubated with DNaseI(DL/Dn), DNA/lipid FBS complex (DLF), DNA/lipid FBS complex incubated with DNaseI (DLF/Dn), DNA/lipid BSA complex (DLB), DNA/lipid BSA complex incubated with DNaseI (DLB/Dn).

**A**

**B**

**C**

**Figure S12.** MTT assay of different gemini lipids and their lipoplexes at various charge ratios along with negative and positive controls, pEGFP-C 3 plasmid and Effectene, respectively. Histograms show cytotoxicity of (A) liposomal suspensions; (B) lipoplexes (C) DNA alone, Effetene alone and its complex with DNA. Experiments were performed in 10% FBS using 0.1 µg of pEGFP-C3 plasmid/well in 96-well plates.

**Figure S13. BrdU assay of HeLa cells treated with different liposomes and lipoplexes used for transfection studies.** (A) In presence of 10% serum (-FBS+FBS) optimized transfection formulations did not show any significant reduction in cell proliferations while (B) in presence of 50% serum (+FBS+FBS), considerable reduction in cell proliferation was noticed. Experiments were performed in duplicate using 0.8 µg DNA/well in lipoplexes.

**(A)**

**(B)** **(C)**

Effectene

Chol-M

CholHG-D

CholG-D

CholHG-4 ox

CholHG-3 ox

CholHG-2 ox

CholHG-1 ox

Cells only

Effectene

Chol-M

CholHG-D

CholG-D

CholHG-4 ox

CholHG-3 ox

CholHG-2 ox

CholHG-1 ox

Cells only

Effectene

Chol-M

CholHG-D

CholG-D

CholHG-4 ox

CholHG-3 ox

CholHG-2 ox

CholHG-1 ox

Cells only

**Figure S14. Fold transfection efficiency of pEGFP-C3 transfected HeLa cells.** Fluorescence was observed by fluorescence microscopy and quantified using FACS analysis. (A) Fold transfection efficiency; (B) FACS histogram obtained upon transfecting pEGFP-C3 in presence of 50 % FBS (-FBS+FBS) and (C) 50 % FBS (+FBS+FBS).


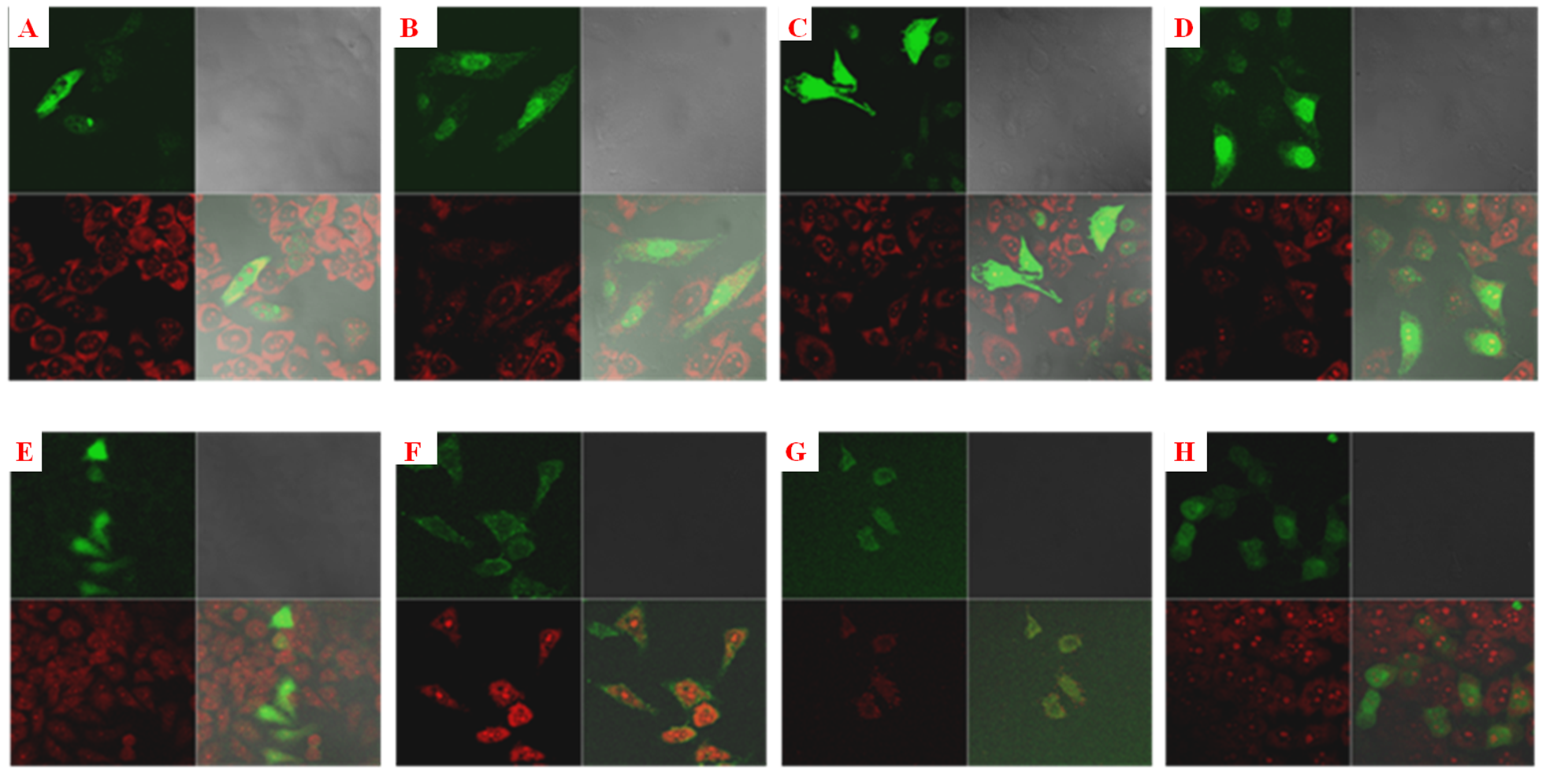


**Figure S15.** **Confocal images of pEGFP-C3 transfected HeLa cells nuclear stained with PI.** HeLa cells transfected with (A) CholHG-1ox:DOPE (1:1) in absence of serum (-FBS-FBS); (B) CholHG-1ox:DOPE (1:1) in 10% serum (-FBS+FBS); (C) Effectene (1:25) in absence of serum (-FBS-FBS); (D) Effectene (1:25) in 10% (-FBS+FBS); (E) CholHG-1ox:DOPE (1:1) in 10% serum (-FBS+FBS); (F) CholHG-1ox:DOPE (1:1) in 50% serum (-FBS+FBS); (G) CholHG-3ox:DOPE (1:1) in 10% serum (-FBS+FBS) and (H) CholHG-3ox:DOPE (1:1) in 50% serum (-FBS+FBS).

**Figure S16. Abstract Figure.** Cholesterol based gemini lipid **CholHG-1ox** possessing -CH_2_-CH_2_-OH at the headgroups and one oxyethylene spacer is at least three times better transfecting agent *in vitro* than one of the best-known commercially available transfecting agents, Effectene (Eff.), in presence of high serum levels (50%).
